# Cross comparison and prognostic assessment of breast cancer multigene signatures in a large population-based contemporary clinical series

**DOI:** 10.1038/s41598-019-48570-x

**Published:** 2019-08-21

**Authors:** Johan Vallon-Christersson, Jari Häkkinen, Cecilia Hegardt, Lao H. Saal, Christer Larsson, Anna Ehinger, Henrik Lindman, Helena Olofsson, Tobias Sjöblom, Fredrik Wärnberg, Lisa Ryden, Niklas Loman, Martin Malmberg, Åke Borg, Johan Staaf

**Affiliations:** 1 Division of Oncology and Pathology, Department of Clinical Sciences Lund, Lund University, Medicon Village, SE 22381 Lund, Sweden; 2 Division of Translational Cancer Research, Department of Laboratory Medicine, Lund University, SE 22381 Lund, Sweden; 3 Division of Clinical Genetics and Pathology, Department of Laboratory Medicine, SE 22185 Lund, Sweden; 4 Department of Immunology, Genetics and Pathology, Uppsala University, SE 75185 Uppsala, Sweden; 5 Department of Clinical Pathology, Uppsala University Hospital, SE 75185 Uppsala, Sweden; 6 Department of Surgical Sciences, Uppsala University, SE 75185 Uppsala, Sweden; 7 Division of Surgery, Department of Clinical Sciences, Lund University, SE 22185 Lund, Sweden; 8 Department of Hematology, Oncology and Radiation physics, Skåne University Hospital, SE 22185 Lund, Sweden

**Keywords:** Breast cancer, Transcriptomics, Breast cancer

## Abstract

Multigene expression signatures provide a molecular subdivision of early breast cancer associated with patient outcome. A gap remains in the validation of such signatures in clinical treatment groups of patients within population-based cohorts of unselected primary breast cancer representing contemporary disease stages and current treatments. A cohort of 3520 resectable breast cancers with RNA sequencing data included in the population-based SCAN-B initiative (ClinicalTrials.gov ID NCT02306096) were selected from a healthcare background population of 8587 patients diagnosed within the years 2010–2015. RNA profiles were classified according to 19 reported gene signatures including both gene expression subtypes (e.g. PAM50, IC10, CIT) and risk predictors (e.g. Oncotype DX, 70-gene, ROR). Classifications were analyzed in nine adjuvant clinical assessment groups: TNBC-ACT (adjuvant chemotherapy, n = 239), TNBC-untreated (n = 82), HER2+/ER− with anti-HER2+ ACT treatment (n = 110), HER2+/ER+ with anti-HER2 + ACT + endocrine treatment (n = 239), ER+/HER2−/LN− with endocrine treatment (n = 1113), ER+/HER2−/LN− with endocrine + ACT treatment (n = 243), ER+/HER2−/LN+ with endocrine treatment (n = 423), ER+/HER2−/LN+ with endocrine + ACT treatment (n = 433), and ER+/HER2−/LN− untreated (n = 200). Gene signature classification (e.g., proportion low-, high-risk) was generally well aligned with stratification based on current immunohistochemistry-based clinical practice. Most signatures did not provide any further risk stratification in TNBC and HER2+/ER– disease. Risk classifier agreement (low-, medium/intermediate-, high-risk groups) in ER+ assessment groups was on average 50–60% with occasional pair-wise comparisons having <30% agreement. Disregarding the intermediate-risk groups, the exact agreement between low- and high-risk groups was on average ~80–95%, for risk prediction signatures across all assessment groups. Outcome analyses were restricted to assessment groups of TNBC-ACT and endocrine treated ER+/HER2−/LN− and ER+/HER2−/LN+ cases. For ER+/HER2− disease, gene signatures appear to contribute additional prognostic value even at a relatively short follow-up time. Less apparent prognostic value was observed in the other groups for the tested signatures. The current study supports the usage of gene expression signatures in specific clinical treatment groups within population-based breast cancer. It also stresses the need of further development to reach higher consensus in individual patient classifications, especially for intermediate-risk patients, and the targeting of patients where current gene signatures and prognostic variables provide little support in clinical decision-making.

## Introduction

Breast cancer is the most common malignancy in women worldwide^1^. It is a heterogeneous disease at the molecular level that translates into diverse clinical manifestation, patient management, therapy options, and ultimately patient outcome. Breast cancer survival has improved greatly over the last decades due to improved screening programs, surgery and adjuvant therapy. However, a therapeutic ceiling may seem to have been reached in terms of curability with approximately 30% of patients eventually relapsing despite recommended adjuvant treatment^2^. The heterogeneity in clinical outcome for patients with similar a priori prognostic variables proposes that, irrespective of treatment, additional tools are required to improve patient stratification, prognostication, and prediction of response to therapy in primary breast cancer.

Gene expression profiling has been used since the early 2000s to stratify early stage breast cancer into molecularly driven subsets associated with patient outcome and specific clinicopathological characteristics. Some studies have resulted in commercial multigene expression profiling tests that can guide physicians in tailoring treatment decisions for individual patients^3^. Two main types of gene expression classifiers have evolved, one aimed at defining subtypes or gene expression phenotypes (GEPs), and one comprised of prognostic or predictive risk predictors (RPs). For both types, a trained classification model (hereafter referred to as a classifier) based on gene expression characteristics of individual samples is used to divide patients into two or more subtypes (GEPs) or risk groups (RPs). Gene signature classifiers may allow for clinically useful disease stratification independent of current prognostic variables, pending adequate validation and clinical implementation. Three RP examples are the Oncotype DX^®^, MammaPrint^®^ and Prosigna^®^ assays that have been validated in large prospective trials and are now offered as commercial tests, of which some are recommended in national and international guidelines (see e.g.^4–10^).

While gene signatures can be assessed and validated in thousands of publicly available breast cancer gene expression profiles (see e.g.^11–13^) and even via online tools^14,15^, there remains a gap in the validation of many signatures in clinical treatment groups in large cohorts of unselected, population-based, primary breast cancer receiving current standard of care therapy. In the current study, we aimed to address this gap by analyzing classification proportions and patient outcome associations of 19 different GEP and RP type gene signatures within a 3520-sample consecutive observational cohort of resectable primary breast cancers from south Sweden. The primary aim was to assess the association with overall survival (OS) for tested gene signatures in nine relevant *clinical assessment groups*. The secondary aim of the study was to describe the classification proportions and classification consensus of the gene signatures in these clinical assessment groups.

## Methods

### Ethics approval and consent to participate

Patients included in this study was enrolled in the Sweden Cancerome Analysis Network - Breast (SCAN-B) initiative^16,17^ (ClinicalTrials.gov ID NCT02306096), approved by the Regional Ethical Review Board in Lund, Sweden (Registration numbers 2009/658, 2010/383, 2012/58, and 2013/459). All patients provided written informed consent prior to study inclusion. All analyses were performed in accordance with patient consent and ethical regulations and decisions.

### Patient material

**Figure 1:**
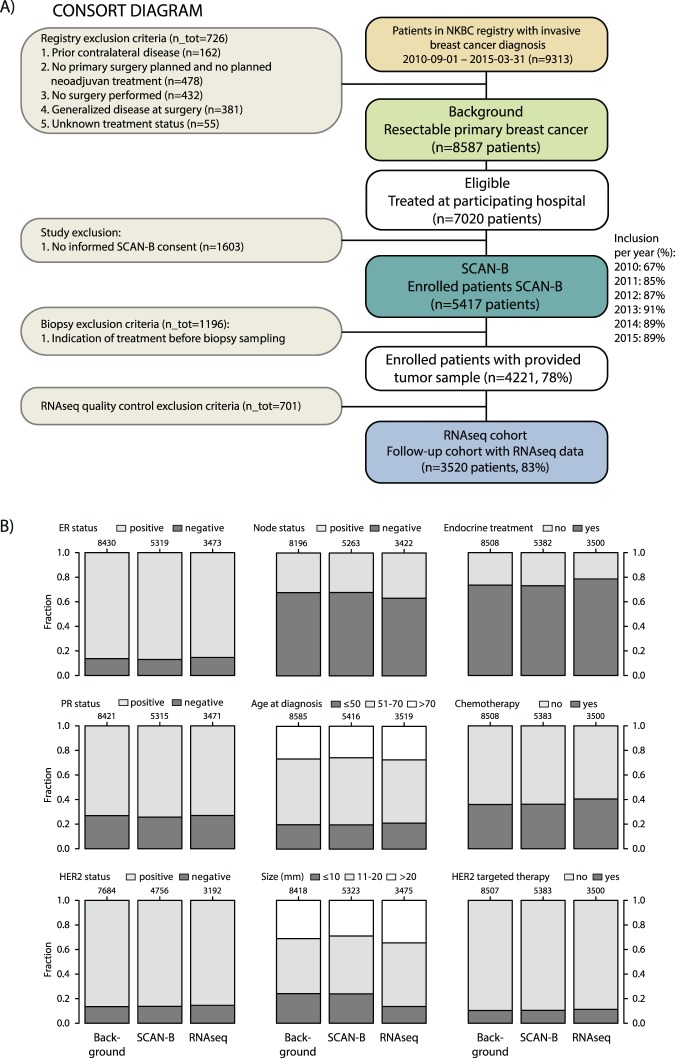
Consort diagram of patient selection and population-based representativeness. (**A**) Consort diagram of patient inclusion. (**B**) Population representativeness for the selection process and final RNAseq cohort illustrated by proportional bar charts for important clinicopathological variables in breast cancer. For each variable, the three bars correspond to the background population (left), enrolled SCAN-B patients (center), and SCAN-B patients with RNA-seq (right) NKBC: Swedish national breast cancer quality registry.

Out of 5417 patients enrolled in SCAN-B between September 1 2010 and March 31 2015 with primary invasive disease, 4221 had primary surgery without neoadjuvant treatment and with a fresh tumor sample taken by a pathologist during the clinical diagnostic routine. Patients were recruited from the following hospitals through sites participating in SCAN-B during the time period: Lund, Malmö, Helsingborg, Kristianstad, Växjö, Halmstad, Uppsala, Karlskrona, Varberg, and Ljungby. 3520 patients were identified with high quality RNA sequencing (RNAseq) data as outlined in Fig. 1. Population-based representativeness for each step in the patient selection process was assessed through comparison between: i) the general comparable breast cancer population in the catchment region (n = 8587), ii) the subset of enrolled SCAN-B patients (n = 5417), and iii) the RNAseq cohort subset (n = 3520). Comparisons were done using clinicopathological data from the Swedish national breast cancer quality registry (NKBC) (Fig. 1). Patient characteristics and clinicopathological variables are described in Table 1, and are according to current clinical definitions in Sweden. Fresh collected tumor samples preserved in RNAlater (Qiagen, Hilden, Germany) were obtained as per established SCAN-B protocols integrated in routine clinical practice and performed by clinical pathologists in regional pathology departments throughout the healthcare region^16,17^. RNA was extracted using the Qiagen Allprep extraction kit (Qiagen, Hilden, Germany) as described^16,17^.

**Table 1:** Patient characteristics and clinicopathological variables of the study cohort based on national cancer quality registry data.

	All samples	ER+/HER2−/LN+	ER+/HER2−/LN−	HER2+/ER+	HER2+/ER−	TNBC
Number of samples	3520	943	1527	321	140	340
**ER status (%)**						
Negative (ER IHC ≤ 10%)*	14.5	0	0	0	100	100
Positive (ER IHC > 10%)*	84.2	100	100	100	0	0
NA	1.3	0	0	0	0	0
**PR status (%)**						
Negative (PR IHC ≤ 10%)*	26.5	13	12	32	96	100
Positive (PR IHC > 10%)*	72.1	87	88	68	4	0
NA	1.4	0	0	0	0	0
**HER2 status (%)**						
Negative	82.4	100	100	0	0	100
Positive	13.2	0	0	100	100	0
NA	4.4	0	0	0	0	0
Age (median and range)	65 (25–100)	65 (25–95)	65 (30–100)	63 (30–95)	70 (35–95)	65 (30–95)
Tumor size (median mm and range)	17 (0–126)	20 (0–126)	15 (1–110)	18 (1–120)	20.5 (0–70)	20 (0–100)
**NHG (%)**						
1	15	13	24	2	0	1
2	47	56	55	30	11	11
3	36	30	20	66	84	84
NA	2	2	1	3	4	3
**Lymph node status (%)**						
0 positive nodes	59	0	100	54	43	64
1–3 positive nodes	27	71	0	27	31	24
≥4 positive nodes	9	23	0	12	19	8
Sub micro metastasis	2	6	0	3	2	2
NA	3	0	0	4	6	2
**Treatment (%)**	**All samples**	**ER+/HER2−/LN+**	**ER+/HER2−/LN−**	**HER2+/ER+**	**HER2+/ER−**	**TNBC**
Endocrine only	49	**48**	**71**	17	1	1
ACT only	8	1	0	0	1	**70**
Endocrine & ACT	22	**49**	**15**	2.5	0	1
Anti-HER2 & ACT	3	0	0	2	**79**	1
Anti-HER2 & ACT & endocrine	8	0.7	0	**74**	6	0
Untreated	10	1.6	**13**	3	8	**24**
NA or other combination	0	0	1	1.5	5	3

*In Sweden the cut-off by IHC for ER and PR is ≤10% staining intensity.

NHG: Nottingham grade index.

ACT: Adjuvant chemotherapy.

Anti-HER2: HER2-targeted therapy (mainly trastuzumab).

NA: data not available.

**Bold** groups indicate clinical treatment groups used for signature evaluation.

### Gene expression analysis

Gene expression profiling of the 3520 patients were performed using RNA sequencing as described^16,18^. Gene expression data is available through Gene Expression Omnibus^19^ (GEO) series GSE96058. Expression data (Fragments Per Kilobase per Million reads, FPKM) was extracted for each case. Nineteen gene classifiers, originating from 15 public gene classifiers, representing different subtype predictors or prognostic predictors were used to classify samples as described in detail in Supplementary Methods using either described/provided algorithms from studies, or implementations available in existing R packages, e.g., genefu^20^ (Table 2). A complete list of classifications for each sample and signature is available as Supplementary Table S1 together with patient characteristics and survival data.

**Table 2:** Gene signatures used for classification.

Signature	Reference	Type	Developed for
1. PAM50 – AIMS	^23^	GEP	All breast cancer
2. PAM50*	^38^	GEP	All breast cancer
3. IC10	^11, 12^	GEP	All breast cancer
4. CIT	^27^	GEP	All breast cancer
5. TNBCtype	^28, 46^	GEP	TNBC
6. HDPP	^43^	GEP	HER2+ breast cancer
7. SDPP	^42^	GEP	All breast cancer
8. SCMOD2	^34^	GEP	All breast cancer
9. GGI	^39^	RP	All breast cancer
10. Gene70	^47^	RP	All breast cancer
11. Oncotype DX	^7^	RP	ER+ breast cancer
12. Gene76	^48^	RP	LN-negative breast cancer
13. Endopredict	^49^	RP	ER+/HER2− breast cancer
14. Genius	^37^	RP	All breast cancer
15. ROR-S*	^38^	RP	All breast cancer
16. ROR-P*	^38^	RP	All breast cancer
17. ROR-T*	^38^	RP	All breast cancer
18. ROR-PT*	^38^	RP	All breast cancer
19. ROR-Tot*	^8^	RP	All breast cancer

*Implementation was performed by using a reference set similar in composition to Parker *et al*.^38^ for gene centering.

GEP: Gene expression phenotype signature.

RP: Risk prediction signature.

Gene set activation status, activated (1), repressed (−1), or latent (NA) for molecular processes was determined using absolute inference of patient signatures (AIPS) models^21^. Activation status from available AIPS models for gene ontology gene sets derived from the Biological Process or Molecular Function ontology^22^ was used to cluster tumors using the R package pheatmap with manhattan based distance and complete linkage.

### Survival analysis

Survival analyses were performed in R version 3.3.0 using the survival package with overall survival (OS) as endpoint. OS was the only endpoint time variable with full coverage for SCAN-B patients at the time of this study. Survival curves were compared using Kaplan-Meier estimates and the log-rank test. Hazard ratios were calculated through univariable or multivariable Cox regression using the *coxph* R function. In multivariable analyses tumor size (mm), patient age at diagnosis, lymph-node (LN) status (node-positive/node-negative), and tumor grade were included as covariates. The full available follow-up time was used in calculations.

## Results

### A population-based breast cancer cohort for representative real-world follow-up analysis

The study cohort is derived from the complete background population of 8587 patients diagnosed with primary resectable breast cancer in the wider catchment region during September 1 2010 and March 31 2015. A total of 5417 cases from the background population were enrolled in the SCAN-B study (Fig. 1), of which 3520 had quality-controlled RNAseq data available. Patient demographics for the SCAN-B study cohorts were evaluated based on national breast cancer quality registry data and found to be highly similar to the background population (Fig. 1B). One exception was a lower proportion of small node-negative tumors in the RNAseq cohort due to scarcity of tissue left after routine clinical diagnostics (Fig. 1B). Together, this assures the approximation of the SCAN-B RNAseq cohort as a good representation of population-based contemporary breast cancer in the South Swedish Healthcare region (comprising ~1.8 million inhabitants) and suitable for making general inferences regarding, e.g., classification proportions and outcome.

Of the 3520 cases with RNAseq, 34% had ≥5 years of follow-up (OS), 23% 4–5 years, 24% 3–4 years, and 16% 2–3 years at the time of this study. Patients were stratified into nine clinical groups based on administered adjuvant treatment and relevant clinicopathological variables. These groups are referred to as *clinical assessment groups* herein and include: i) triple negative breast cancer (TNBC) with adjuvant chemotherapy (ACT, n = 239), ii) adjuvantly untreated TNBC (n = 82, only treatment was surgery), iii) HER2+/ER− with adjuvant anti-HER2+ ACT treatment (n = 110), iv) HER2+/ER+ with adjuvant anti-HER2+ACT + endocrine treatment (n = 239), v) ER+/HER2−/LN− with adjuvant endocrine treatment (n = 1113), vi) ER+/HER2−/LN− with adjuvant endocrine + ACT treatment (n = 243), vii) ER+/HER2−/LN+ with adjuvant endocrine treatment (n = 423), viii) ER+/HER2−/LN+ with adjuvant endocrine + ACT treatment (n = 433), and ix) adjuvantly untreated ER+/HER2−/LN− (n = 200, only treatment was surgery) (Table 1, highlighted fields).

### Subtype proportions of 19 gene signatures in population-based breast cancer

**Figure 2:**
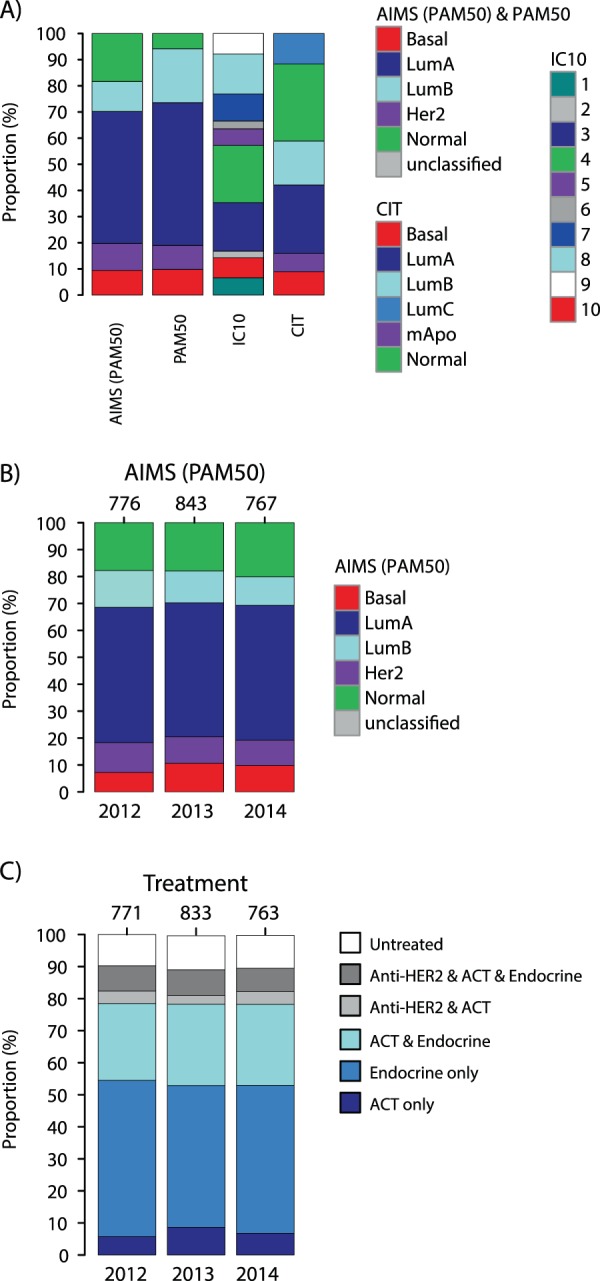
Molecular subtypes and administered therapy for the study cohort. (**A**) Proportions of subtypes from four molecular subtype signatures in the complete RNAseq cohort. (**B**) Subtype proportions of the AIMS-PAM50 single sample predictor for patients diagnosed 2012, 2013, and 2014 respectively, illustrating the stability of the underlying patient demographics across inclusion years. (**C**) Treatment proportions according to national registry data for RNAseq patients diagnosed 2012, 2013, and 2014 respectively, illustrating the stability of the underlying patient demographics during inclusion years. ACT: adjuvant chemotherapy. Endocrine: endocrine treatment.

The 3520 tumors were classified according to 19 gene signatures (Table 2), as detailed in Supplementary Methods. Subtype proportions across all 3520 samples for four molecular subtype classifiers (AIMS-PAM50, PAM50, CIT, and IC10) in breast cancer are shown in Fig. 2A. To further verify the generalizability of the population-based cohort with respect to classifications, we stratified classifications by the AIMS-PAM50 single sample predictor (which is independent of cohort centering and thus insensitive to cohort composition bias) by year of diagnosis (2012, 2013, 2014). As seen in Fig. 2B, subtype proportions for enrolled patients were highly similar across inclusion years. Correspondingly, proportions of administered treatment regime were also consistent across year of diagnosis (Fig. 2C). Together, these results support that classification findings and outcome results made in this cohort may be generalized to future patient populations of similar demographic composition.

**Figure 3:**
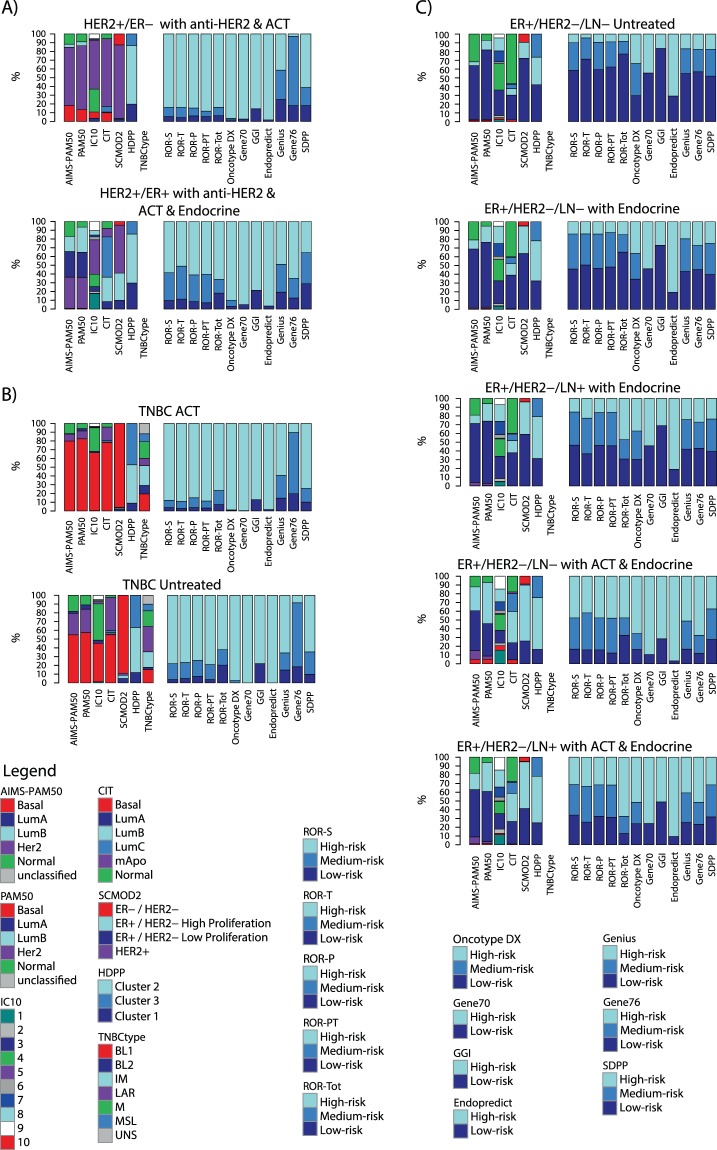
Gene signature class proportions across nine clinical assessment groups in population-based breast cancer. (**A**) HER2+ disease stratified into two clinical assessment groups: i) HER2+/ER− with anti-HER2 and adjuvant chemotherapy (ACT), and ii) HER2+/ER− with anti-HER2, adjuvant chemotherapy (ACT) and endocrine therapy. (**B**) TNBC disease stratified into two clinical assessment groups: i) TNBC with adjuvant chemotherapy, and ii) untreated TNBC. (**C**) ER+/HER2− disease stratified by lymph-node status (lymph-node negative: LN−, positive: LN+) and adjuvant therapy into five clinical assessment groups: (i) ER+/HER2−/LN− untreated, (ii) ER+/HER2−/LN− with endocrine therapy only, (iii) ER+/HER2−/LN+ with endocrine therapy only, (iv) ER+/HER2−/LN− with adjuvant chemotherapy (ACT) and endocrine therapy, and (v) ER+/HER2−/LN+ with adjuvant chemotherapy (ACT) and endocrine therapy.

Classification proportions for all signatures across the nine clinical assessment groups are shown in Fig. 3 and Supplementary Table S2. Reassuringly, predicted class assignment of reported low-risk and high-risk groups match, in general, the corresponding clinical management of the patients according to Swedish national breast cancer quality registry data. For ER+/HER2− patients receiving only adjuvant endocrine treatment or no adjuvant treatment, risk-classifiers such as the Oncotype DX, the 70-gene signature (Gene70), ROR-variants, and GGI (genomic grade index) mainly predicted cases as low-risk or medium/intermediate risk (approximately 40–80%) (Fig. 3C). The Endopredict signature was an exception, with nearly 80% high-risk classified samples in these assessment groups. In contrast, among ER+/HER2− patients receiving additional adjuvant chemotherapy, a minority was predicted as low-risk, consistent with, e.g., a higher proportion of Luminal B cases in such groups. Also, for HER2− positive and TNBC patients (Fig. 3A,B) most RP classifiers predicted only a small number of cases as low-risk. The proportional analyses also demonstrate the heterogeneity in clinical assessment groups defined by IHC within TNBC, ER+/HER2−, and HER2-positive disease with respect to gene expression subtypes with all molecular subtypes represented, albeit at varying degree depending on assessment group. For instance, while relatively high heterogeneity was observed in TNBC and HER2+/ER+ disease without adjuvant treatment, considerably lower heterogeneity was observed among TNBC and HER2+/ER− tumors administered chemotherapy (approximately 70–80% were basal-like or HER2-enriched tumors, respectively). While these subtype differences in part represent underlying biology, it should be noted that this observation is likely also driven by the inherent problems in classification by nearest centroids as aptly illustrated previously for PAM50^23^.

### Gene signature associations with patient outcome in clinical breast cancer subgroups

**Figure 4:**
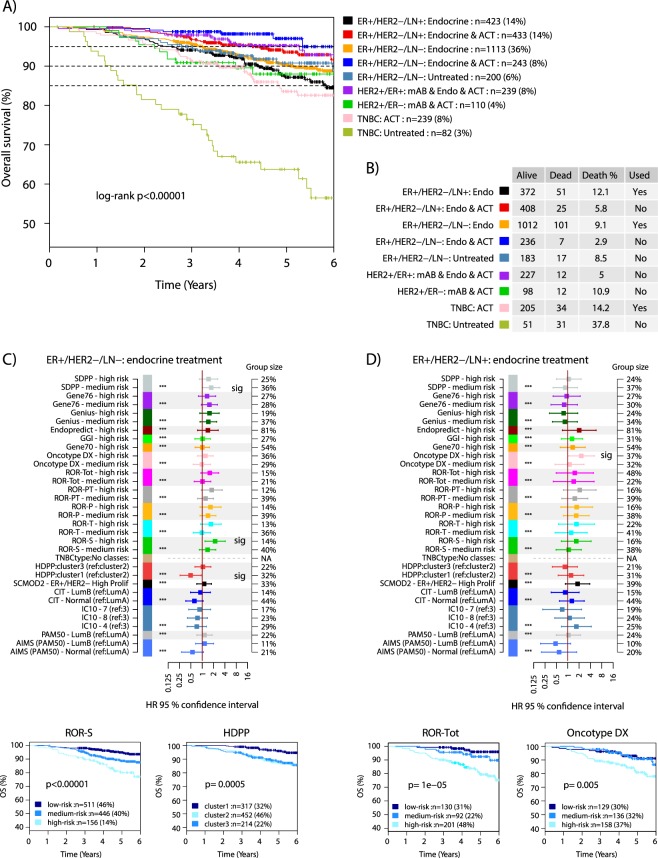
Outcome analyses for clinical assessment groups and gene signatures in ER+/HER2− disease. (**A**) Kaplan-Meier plot of OS for the nine clinical assessment groups using all available samples. Percentages in parentheses represent proportion of entire cohort. (**B**) Table of events per clinical assessment group, outlining the number of cases with events, and a note on whether a group is kept for subsequent outcome analysis. (**C**) Forest plot of hazard ratios (HR) with 95% confidence interval for each signature class from multivariable Cox regression analysis using tumor size, patient age, lymph node status (where applicable), and tumor grade as covariates in the 1113 ER+/HER2−/LN− tumors with endocrine treatment only. Signature classes smaller than 8% of the total population are excluded from multivariable analysis. If not otherwise stated, the reference group is the low-risk group for a signature. Significant classes marked (sig). Bottom: selected Kaplan-Meier plots for the ROR-S and HDPP signatures in these cases. (**D**) Similar forest plot as in C but for the 423 ER+/HER2−/LN+ tumors with endocrine treatment only. Significant classes marked (sig). Bottom: selected Kaplan-Meier plots for the ROR-Tot and Oncotype DX signatures in these cases. * indicates significance level of a likelihood ratio test. ACT: adjuvant chemotherapy. Endo: endocrine treatment. mAB: anti-HER2 blockade. P-values in Kaplan-Meier plots were calculated using the log-rank test.

Across all patients, the nine clinical assessment groups were significantly associated with differences in OS (Fig. 4A). Observed differences in OS are to a great extent explained by the clinical management and patient demographics (e.g. patient age) of the assessment groups. Thus, gene signatures need to be evaluated and compared for prognostic value within the specified assessment groups. Six of the nine clinical assessment groups were deemed unsuited for prognostic evaluation at this time (Fig. 4B). Group exclusion was due to combinations of group size, low number of events (recorded deaths), or bias in age (untreated TNBC) not compatible with overall survival as endpoint. The low number of events is mainly attributed to the short follow-up time that accompany a contemporary cohort with patients diagnosed 2010–2015 and subjected to modern clinical management including extensive HER2 blockade. For ER+ disease, the follow-up time is relatively short considering the nature of late recurrences in this subgroup of breast cancer with modern therapy^24^. After group exclusion, three groups remained for further outcome analyses: i) ER+/HER2−/LN− tumors with endocrine treatment, ii) ER+/HER2−/LN+ tumors with endocrine treatment, and iii) TNBC with adjuvant chemotherapy.

**Figure 5:**
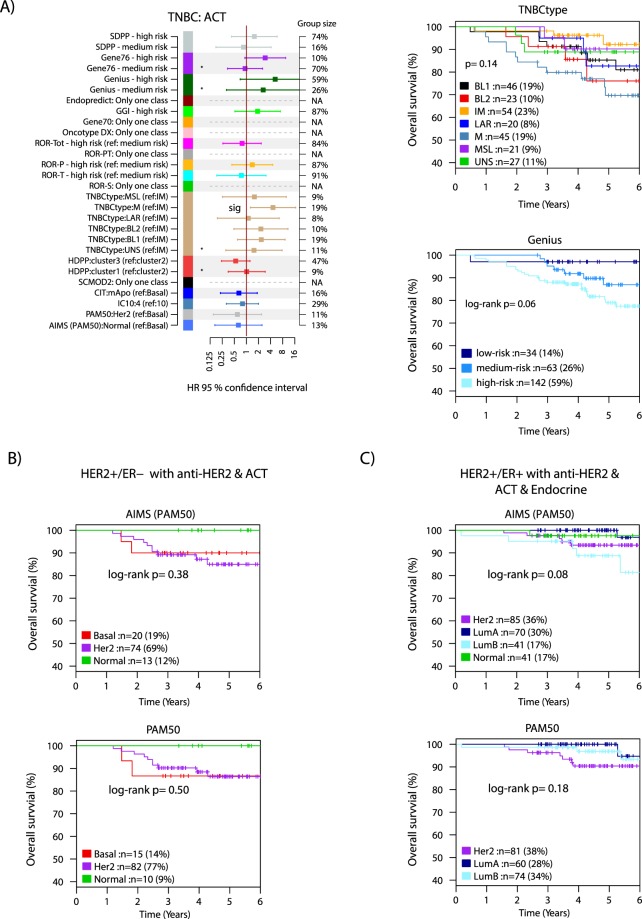
Outcome analyses in TNBC and HER2+ disease. Signature classes <8% are excluded from multivariable analysis. If not stated, the reference group is the low-risk group for a signature. (**A**) Forest plot displaying hazard ratios (HR) with 95% confidence interval for respective signature class from multivariable Cox regression analysis using tumor size, patient age, lymph node status, and tumor grade as covariates in the 239 TNBC tumors with adjuvant chemotherapy. Significant classes marked (sig). For several signatures only one class existed, thus no values were calculated. Right: selected Kaplan-Meier plots for the TNBCtype and Genius signatures in these cases. (**B**) Kaplan-Meier plots of PAM50 subtypes defined through the single sample predictor AIMS or a centroid-based approach in HER2+/ER− disease treated with combined HER2-blocade and adjuvant chemotherapy. (**C**) Kaplan-Meier plots of PAM50 subtypes defined through the single sample predictor AIMS or a centroid-based approach in HER2+/ER+ disease treated with combined HER2-blocade, endocrine therapy, and adjuvant chemotherapy. ACT: adjuvant chemotherapy. Endo: endocrine treatment. P-values in Kaplan-Meier plots were calculated using the log-rank test.

For these three assessment groups we performed Kaplan-Meier analysis, univariable, and multivariable Cox regression modeling for each signature using tumor size (mm), patient age, lymph node status (when applicable), and tumor grade (Nottingham Histological Grade) as covariates (Figs 4C,D and 5A). While many signatures showed significant hazard ratios in univariable analysis (Supplementary Fig. S1) statistical significance in the comparably challenging multivariable analysis (correcting for tumor size, age, and grade) was limited to only a few signatures in the two HER2-negative luminal clinical assessment groups, including SDPP, HDPP and ROR-S in ER+/HER2−/LN− endocrine treated tumors, and Oncotype DX in ER+/HER2−/LN+ endocrine treated tumors (Fig. 4C,D). Importantly, while not reaching statistical significance, many signatures were borderline non-significant in the analyses, likely due to the limited follow-up time (see Supplementary Fig. S2 for all multivariable analyses).

Based on neoadjuvant studies it has been suggested that for HER2+ disease the PAM50 HER2-enriched molecular subtype could be associated with a higher complete response rate (a surrogate end-point for long term outcome) to HER2-blockade^25,26^. While not possible to fully evaluate this in multivariable analysis, we performed Kaplan-Meier analysis of the PAM50 subtypes versus OS for subtypes defined by either the AIMS-PAM50 single sample predictor, or through a more conventional centroid-based approach in HER2+ disease stratified by ER-status. We did not observe any support for tumors of the HER2-enriched subtype having a better OS after HER2-blockade + additional adjuvant treatments in our population-based series in neither HER2+/ER− nor HER2+/ER+ disease (Fig. 5B-C).

### Consensus of risk prediction signatures

Class proportions for the tested gene signatures (Fig. 3) demonstrate that, within clinical assessment groups, many gene signatures produce overall similar proportions of classification (e.g. risk predictions in HER2-negative luminal subgroups, Fig. 3C). Also, whereas risk prediction signature classification is in general well aligned with clinical assessment groups, the class proportions reveal that contrasting classification do exist. For example, the low-risk clinical assessment group of untreated ER+/HER2− disease does not exclusively display low-risk signature classes.

**Figure 6:**
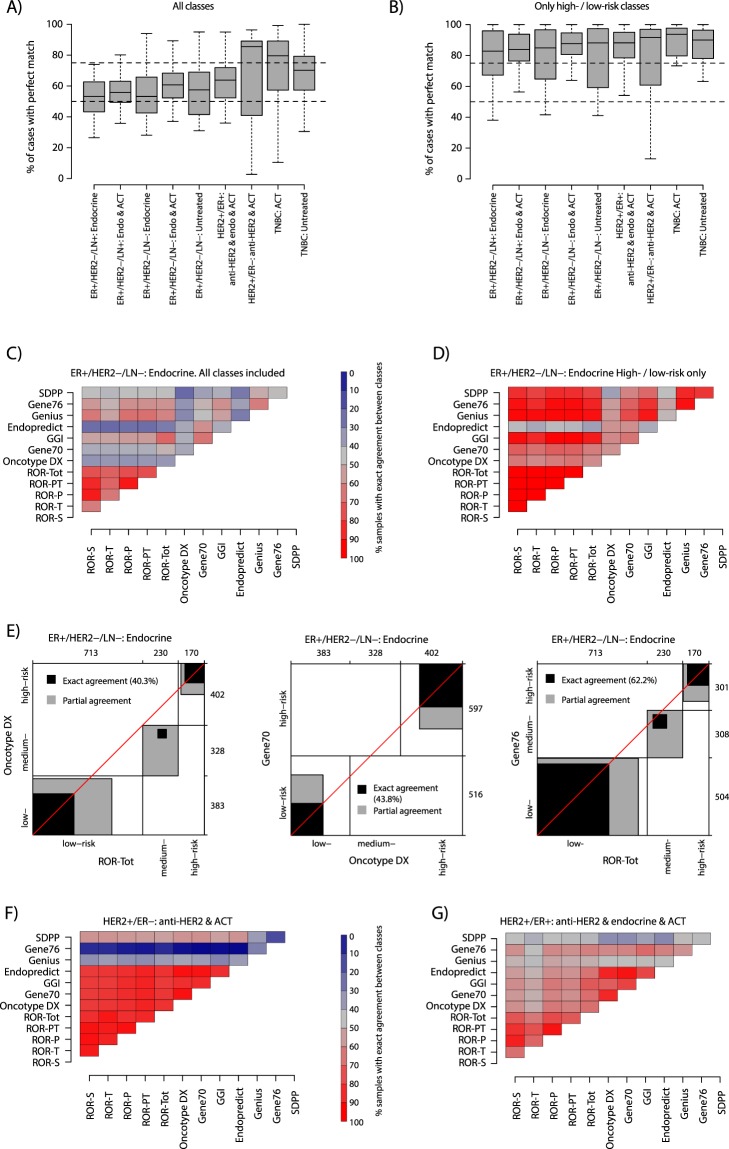
Signature class consensus for risk prediction signatures. (**A**) Distribution of exact agreements between risk prediction signature pairs summarized by clinical assessment group. Analyzed risk prediction signatures include ROR variants, Oncotype DX, Gene70, GGI, Endopredict, Genius, Gene76 and SDPP. For each compared signature pair the exact classification agreement using all available classes (low-, medium/intermediate-, and high-risk) was calculated. Next, all agreement values from all signature combinations were summarized into a box plot for each assessment group. (**B**) Same analysis as in A, but now only for comparisons after omitting medium/intermediate-risk classified samples. I.e., all patients with a medium/intermediate-risk prediction in a signature pair comparison were omitted before calculating the exact agreement. (**C**) Percentage of exact risk class agreement for risk prediction signature pairs in ER+/HER2−/LN− endocrine treated samples using all available signature classes in the individual comparisons. The heatmap corresponds to all values included in the corresponding assessment group box plot in A. (**D**) Similar display as in C, both now for low-risk and high-risk classified samples only in ER+/HER2−/LN− endocrine treated patients. In this analysis, all patients with a medium/intermediate-risk prediction in a signature pair comparison were omitted before calculating the exact agreement. (**E**) Specific agreement charts for Oncotype DX versus ROR-Tot (left), Gene70 versus Oncotype DX (center), and Gene76 versus ROR-Tot (right) in ER+/HER2−/LN− endocrine treated samples similar as described^32^. Briefly, rectangles are drawn for each level of the test outcomes, i.e., low-, medium/intermediate-, and high-risk, based on the row and column cumulative totals. The boundaries of the rectangles along both axes represent the number of tumors categorized as that outcome group for each test. Black squares within the rectangles represent exact agreement between the levels of the two tests and are of size based on the cell frequencies and located according to the cumulative totals of the previous levels. Gray rectangles represent partial agreement, where the scores from one test are within one level of those from the other test, i.e., a low-risk prediction in one test but medium/intermediate-risk in the other test. White areas within the rectangle reflect disagreement by more than one level, i.e., low-risk in one test and high-risk in the other test. (**F**) Percentage of exact risk class agreement for risk prediction signature pairs in the HER2+ER− assessment group using all available signature classes in the individual comparisons. (**G**) Percentage of exact risk class agreement for risk prediction signature pairs in the HER2+ER+ assessment group using all available signature classes in the individual comparisons. ACT: adjuvant chemotherapy. Endo: endocrine treatment.

However, the class proportion results within assessment groups do not resolve specific signature classification concordance for individual tumors. To more thoroughly study consensus we focused on the risk prediction (RP) signatures, as these provide a straightforward comparison of low-, medium/intermediate- and high-risk class, and cross-comparisons of different subtyping schemes have been reported previously^12,23,27,28^. For the RP signatures (ROR variants, Oncotype DX, Gene70, GGI, Endopredict, Genius, Gene76 and SDPP) we first summarized the pairwise percentage of exact agreement between signature predictions using available classes (low-, medium/intermediate-, and high-risk, Fig. 6A), or using only low- and high-risk classes (thus excluding all samples with an medium/intermediate risk class, Fig. 6B) across all clinical assessment groups. This analysis revealed that for all pairwise agreements using all available classes, between 50–60% of samples on average in ER+ assessment groups had exact prediction agreement (Fig. 6A). For ER-negative assessment groups the average was higher. When using only low- and high-risk samples the average was consistently higher in all groups (between 80–95%, Fig. 6B). To illustrate these findings we created detailed agreement maps for each assessment group using all available cases (example in Fig. 6C, all groups in Supplementary Fig. S3) or using low- and high-risk, and also detailed agreement plots of individual signature pairs (Fig. 6D). Figure 6C shows that in ER+/HER2−/LN− endocrine treated disease the different ROR variants showed high exact agreement, extending to the majority of clinical assessment groups (Supplementary Fig. S3). We also observed higher exact agreement between the ROR variants, Gene76, GGI, and Genius signatures, while the Endopredict signature showed low agreement with most other signatures (Fig. 6C, Supplementary Fig. S3). When analyzing exact agreement for only low- and high-risk cases in the ER+/HER2−/LN− endocrine assessment group we noted generally high (>60%) to very high agreement (>80%), except for Endopredict (Fig. 6D). The detailed agreement charts for ROR-Tot, Oncotype DX, and Gene70 in Fig. 6E illustrates that discrepant classification is mainly of low- versus medium/intermediate-risk, and high- versus medium/intermediate-risk (gray zones, Fig. 6E) substantiating our general observations in Fig. 6A,B. In the HER2+/ER− and TNBC assessment groups we observed high exact agreement between the majority of signatures, except for Genius and Gene76 (Fig. 6F and Supplementary Fig. S3). This high agreement is mainly due to the fact that most signatures predict these tumors as high-risk (see Fig. 2A,B). For the HER2+/ER+ assessment group generally lower exact agreement was observed, more in line with the ER+/HER2− groups (Fig. 6G).

### Gene signatures, consensus and association with transcriptional programs

**Figure 7:**
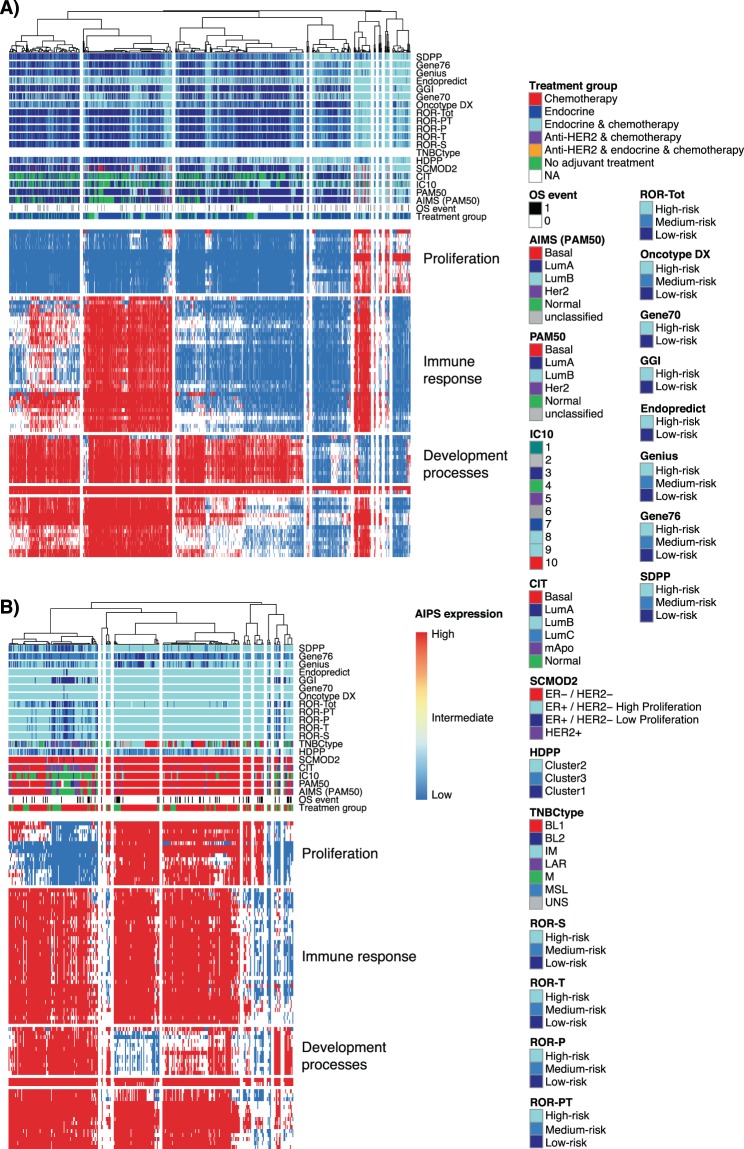
Signature classification consensus and transcriptional programs in clinical assessment groups. AIPS was used to derive activation status of gene signatures related to gene ontology terms. (**A**) Signature classifications and AIPS heatmap for 1563 ER+/HER2−/LN− tumors. (**B**) Signature classifications and AIPS heatmap for 321 TNBC tumors.

To further assess gene signature agreement as well as the clinical assessment groups with respect to molecular function activity, we used a binary-like gene set activity approach, AIPS [19]. We determined activation status from RNAseq data using AIPS models available for gene ontology terms for molecular processes and clustered tumors based on these (Fig. 7 and Supplementary Fig. S4). For ER+/HER2−/LN− and ER+/HER2−/LN+ disease, overall consensus between different risk predictors matches the activation status of gene sets related to proliferation, with the high-risk class coinciding with activation (Fig. 7A, and Supplementary Fig. S4A,B). The results also demonstrate the nature of low-, medium/intermediate-, high-risk classifications for the risk predictors that are clearly associated with gene expression related to proliferation, which in turn, is not strictly dichotomized in two or more separate groups. Heterogeneity within the clinical groups of ER+ disease is also clearly visualized by groups of tumors with essentially the same molecular phenotypes, for instance related to active cell proliferation, found in both the LN− and LN+ subsets and with mixed administered treatment (see Fig. 7A treatment group annotation and Supplementary Fig. S4A,B, respectively). For the molecular subtype signatures in these two clinical groups, it is also evident that high-risk subtypes like luminal B follow the same pattern and coincide with high-risk class predictions and the molecular phenotype of active proliferation.

For TNBC, consensus among risk predictors appears highest for the tumors with activated cell proliferation (Fig. 7B and Supplementary Fig. S4C). This is not surprising considering that cell proliferation is known to be a key prognostic component of most of the initial gene signatures reported in breast cancer, and that cell proliferation is typically high in TNBC. More heterogeneity in the class consensus of both risk predictors and molecular subtype signatures is evident in TNBC tumors without activated cell proliferation. For HER2+ disease the same molecular phenotypes are discernible but there is heterogeneity of risk predictor and molecular subtype classes and less clear association (Supplementary Fig. S4D,E). However, regarding at TNBC and HER2+ breast cancer it should be noted that the majority of initial risk predictor signatures assessed herein were developed and targeted for use in ER+/HER− breast cancer only.

## Discussion

In the current study we have analyzed classification proportions and prognostic impact of 19 gene signatures in contemporary population-based early breast cancer stratified into clinical assessment groups by clinicopathological variables and adjuvant treatment. Studies of the prognostic value of different gene signatures in breast cancer have been reported since more than a decade (see e.g.^13,29–35^). Prior studies have mainly reported on either smaller patient cohorts or from larger meta-analyses involving the merger of heterogeneous publicly available datasets. Agreement in classification has been suggested to be moderate even for commercial tests^31,32,36^. In this context, the novelty and impact of this study lies in the RNAseq analysis of ~3500 consecutive breast cancer patients collected over a period of five years (2010–2015) in a defined geographic region and healthcare region (south Sweden) following contemporary treatment guidelines. Based on a rigorous population-based approach with consistently high inclusion rates due to a seamless integration of patient enrollment and tissue sampling with routine clinical practice in the participating SCAN-B regional hospitals, the current study cohort is clearly representative of the general patient demographics in the region (Figs 1 and 2). Importantly, this unique setting provides a context and support for that overall themes in the results can be extended and generalized to a national or North European breast cancer population.

The primary aim of this study was to investigate the association of 19 different gene signatures with OS in actual clinical assessment groups in population-based breast cancer. We did not aim to identify (select) a winning signature and this study is not designed to do so for a number of reasons, including that treatment in the SCAN-B cohort is not based on randomization. Firstly, for many gene signatures analyzed in this or other studies there are no RNAseq specific classifiers available with appropriately optimized risk stratification cut-offs. We therefore chose to use a straightforward approach to gene signature implementation, based on classification strategies similar to previous studies and occasionally representing mimicked signatures (see e.g.^13,29,33,37^). We acknowledge that more adapted classifier implementations could alter classifications for individual samples. However, the latter was not the focus of this study, and even so, we argue that our results still remain valid due to the comprehensive population-based context, and moreover that an adaptive implementation approach is fraught with its own complications. It is also important to acknowledge that derived classifications are relative to a population, and not to individual patients. Secondly, the current study is limited to the use of overall survival as this was the only clinical endpoint with complete coverage through the Swedish national quality registry for breast cancer at present. Finally, due to the contemporary nature of the SCAN-B initiative, follow-up time for included patients is still relatively short for large subgroups considering the use of modern standard of care therapy, especially for ER+ disease that is characterized by late recurrences.

Despite the above limitations we find that most investigated gene signatures already provide additional prognostic information beyond conventional clinicopathological factors in specific clinical assessment groups, mainly the ER+/HER2− ones (Figs 4, 5 and Supplementary Figs S1–S2). Variants of the risk of recurrence score, ROR, appear to do generally well, in line with recent head to head studies of corresponding commercial signatures^36^. However, we note little difference in classifications and prognostic performance between the simpler and earliest variant, ROR-S^38^, and the most recent ROR-Tot that incorporates tumor size, proliferation as well as optimized risk cut-offs dependent on lymph-node status^8^. It should also be emphasized that the multivariable models used in this study are challenging for any gene signature considering stratification in assessment groups and the covariates used, including tumor grade and tumor size. Tumor grade has repeatedly been tightly linked to tumor cell proliferation^39,40^ that in turn represents a key prognostic component in most early predictive and prognostic breast cancer gene signatures, including the molecular subtypes^13,41^. Thus, despite not reaching formal statistical significance, we expect that the majority of tested signatures will, at least in ER+/HER2− disease treated with adjuvant endocrine therapy, demonstrate independent prognostic value when longer follow-up is available. For clinical assessment groups other than ER+/HER2− disease, less apparent prognostic value was observed for the tested signatures. In part, this may be explained by the fact that the majority of signatures were not developed to specifically target these subgroups. As shown multiple times, these subgroups appear to have other transcriptional programs associated with prognosis than those important in ER+/HER2− disease (e.g.^13,41^). For instance, within the HER2+ assessment groups we did not observe any signs of molecular subtype being associated with better overall survival, in contrast to results suggesting better response in neoadjuvant studies^25,26^. Taken together, our results demonstrate in a population-based breast cancer context, despite certain limitations, that gene expression analysis has independent prognostic information of real clinical value. In a few years’ time, the contemporary SCAN-B cohort will be able to provide more definite answers on gene signature value based on longer follow-up time and the addition of metastasis-free survival as endpoint.

The second aim of this study was to describe gene signature class proportions in breast cancer stratified into actual clinical assessment groups. Due to the population-based setting of our cohort, we argue that the results shown in Fig. 3 are representative of breast cancer in Sweden and likely northern Europe too, and they are not affected by the current limited follow-up time. Here, our results demonstrate that clinicians, using current clinical tools and practice, are generally well aligned with gene signature classifications in identifying patients with different prognosis. For example, the proportion of tumors classified as low-risk is markedly lower in ER+/HER2− breast cancer patients receiving adjuvant chemotherapy as compared to corresponding patients not administered any adjuvant systemic treatment (Fig. 3C). The class proportion and consensus analyses also illustrate that many of the analyzed signatures (e.g. most risk predictors, but also molecular subtyping signatures) provide little potential value to further stratify the clinical groups of TNBC and HER2+ disease. The patient outcome, proportional, and consensus analyses also illustrate the interchangeability of many gene signatures on a patient group level, meaning that it matters less which specific signature that is used, an observation that has been reported previously (e.g.^13,29,32^). However, on an individual patient level the picture appears more complicated with exact agreement between risk prediction signatures ranging between 50–60% on average in ER+ assessment groups when using all prediction classes (low-, medium/intermediate-, high-risk), and with occasional pair-wise comparisons dropping below 30% (Fig. 6A). Bartlett *et al*. recently reported that only ~40% of cases were uniformly classified by four commercial gene signature tests in high-risk ER+/HER2− disease^32^. Our findings support these results for the corresponding ROR-Tot, Oncotype DX, and Gene70 comparisons in ER+/HER2− disease (Fig. 6). In contrast, other signature combinations show clearly higher agreement (e.g. Fig. 6E and Supplementary Fig. S3). Moreover, when omitting medium/intermediate-risk samples from the comparisons, a substantially higher exact prediction agreement was observed in all assessment groups (Fig. 6B and Supplementary Fig. S3), suggesting that the prediction of medium/intermediate-risk cases represent the greatest source of variation between signatures (at least for 3-class RP signatures). Three conclusions from these observations are: i) dedicated gene signatures for non-ER+/HER2− clinical subgroups are needed, of which a few have indeed been reported (e.g.^42,43^ in this study) but none are in clinical use, ii) robust classifiers are needed, perhaps in the form of “true” single sample classifiers or mixed predictors (e.g. involving scores instead of current binary classes and with additional integration of clinicopathological variables), and iii) consensus signature voting may yield better confidence in classification results. To explore the latter assumption, we created voted risk predictions based on exact agreement between seven or eight specific RP signatures, showing that these voted predictions in ER+ disease defines a low-risk subgroup 7–19% in size with >95% overall survival in the ER+/HER2− clinical assessment groups (Supplementary Fig. S5). Obviously, consensus risk voting favors global transcriptome analyses such as RNAseq over single signature tests through the possibility to derive any number of different signatures from the same sample data.

For the first conclusion, the AIPS analysis of activated gene signatures illustrates the heterogeneity within clinical assessment groups (Fig. 7 and Supplementary Fig. S4). Even though the observed misalignment only covers part of the cases, it suggests that further refinement of clinical assessment groups and even molecular subtyping schemes (for instance PAM50) is feasible and can complement current practice to improve disease stratification. It is also clear that other transcriptional profiles, e.g. involving immune characterization, as well as mutational signatures may complement clinically relevant stratification of HER2+ and TNBC. An example of the latter is the development of specific whole genome sequencing (WGS) based tools for prediction of *BRCA1* and *BRCA2* mutation status^44,45^. This represents an approach of defining novel patient subgroups based on genetic phenotypes that may be targetable in a similar way as in hereditary cancer.

Based on the availability of large population-based RNAseq cohorts like the SCAN-B initiative, current and new signatures may be developed with respect to a more general contemporary breast cancer population. We have demonstrated the possibility to enroll large cohorts of patients and generate RNAseq data in real-time for possible implementation and use at multidisciplinary tumor board conferences across a wide geography^17^. Combined with existing and new classifiers transferred into single sample proxies, this approach can represent a crucial bridge in bringing gene signatures closer to actual clinical use for early stage breast cancer in the increasingly complex clinical management of the disease.

## Conclusions

Based on analysis of 19 gene signatures in ~3500 consecutive, contemporary breast cancer cases collected in a truly population-based context, our results support the usage of gene expression signatures in specific clinical treatment groups. Our study also stresses the need of further development to reach higher consensus in individual patient classifications and the targeting of the patient subsets where current gene signatures and prognostic variables provide little support in clinical decision-making.

## Supplementary information

Supplementary Information

Dataset 1

## Data Availability

Gene expression data is available through Gene Expression Omnibus^19^ (GEO) series GSE96058. A complete list of classifications for each sample and signature is available as Supplementary Table S1.
