# Supplementary material for: Cross comparison and prognostic assessment of breast cancer multigene signatures in a large population-based contemporary clinical series
Source: Sci Rep. 2019 Aug 21;9:12184. doi: 10.1038/s41598-019-48570-x (PMC6704148; doi:10.1038/s41598-019-48570-x)

Supplementary Methods

Cross comparison and prognostic assessment of breast cancer multigene signatures in a large population-based contemporary clinical series

Johan Vallon-Christersson[1], Jari Häkkinen[1], Cecilia Hegardt[1], Lao H. Saal[1], Christer Larsson[2], Anna Ehinger[1,3], Henrik Lindman[4], Helena Olofsson[4,5],Tobias Sjöblom[4], Fredrik Wärnberg[6], Lisa Ryden[7], Niklas Loman[1,8], Martin Malmberg[1,8], Åke Borg[1,a], Johan Staaf[1,a,*]

[1] Division of Oncology and Pathology, Department of Clinical Sciences Lund, Lund University, Medicon Village, SE 22381 Lund, Sweden
[2] Division of Translational Cancer Research, Department of Laboratory Medicine, Lund University, SE 22381 Lund, Sweden
[3] Division of Clinical Genetics and Pathology, Department of Laboratory Medicine, SE 22185 Lund, Sweden
[4] Department of Immunology, Genetics and Pathology, Uppsala University, SE 75185 Uppsala, Sweden
[5] Department of Clinical Pathology, Uppsala University Hospital, SE 75185 Uppsala, Sweden
[6] Department of Surgical Sciences, Uppsala University, SE 75185 Uppsala, Sweden
[7] Division of Surgery, Department of Clinical Sciences, Lund University, SE 22185 Lund, Sweden
[8] Department of Hematology, Oncology and Radiation physics, Skåne University Hospital, SE 22185 Lund, Sweden

*: Corresponding author
a: These authors contributed equally to the study

Corresponding author:
Johan Staaf, PhD, Associate Professor
Address: Division of Oncology and Pathology, Department of Clinical Sciences Lund, Lund University, Medicon Village, SE 22381 Lund, Sweden
Email: johan.staaf@med.lu.se
Fax: +4646147327
Tel: +46462221444

Patient cohort

Clinical and histopathological data were obtained for SCAN-B [1, 2] (ClinicalTrials.gov ID NCT02306096) patients diagnosed and enrolled between 01/09/2010 to 03/31/2015 from the Swedish national breast cancer quality registry (NKBC).

RNAsequencing

RNAsequencing and initial quality control were performed as described [1, 3]. 3520 patients with quality controlled RNA sequencing data was available. In this study, only RefSeq genes were retained, and data were summarized on gene and summarized to FPKM counts. To all FPKM data an offset of +1 was added, and then the data was log2 transformed before further usage.

Signature classification

Nineteen gene classifiers, originating from 15 public gene classifiers, representing different subtype predictors or prognostic predictors were used to classify samples. Classification was performed as outlined below for each signature:

1. *PAM50-AIMS*. For classification we used the R-package *AIMS* version 1.10.0 with default parameters, using Entrez gene identifiers for mapping. Classification was performed for the entire data matrix (n x m, corresponding to genes x samples).
2. *PAM50*. PAM50 classification was performed by a nearest-centroid implementation using correlation-based distance to subtype centroids from Parker et al. [4]. Before calculating correlations for individual samples, gene expression data was normalized by gene centering against a selected and fixed reference sample set selected to match clinical characteristics of the original training set from Parker et al.
3. *IC10*. For classification we used the R-package *iC10* version 1.1.3. Prior to the classification data was mean-centered across samples. Classification was performed for the entire data matrix (n x m, corresponding to genes x samples). Before the actual iC10() classification function we matched features based on gene identifier through the matchFeatures() function, and normalized using normalizeFeatures(,method="scale") function as described in the examples of the package.
4. *CIT*. For classification we used the R-package *citbcmst* version 1.0.4 with Pearson correlation as distance method. The package centers data itself. Classification was performed for the entire data matrix (n x m, corresponding to genes x samples).
5. *TNBCtype*. For classification we used the web-based tool described by Chen et al. [5]. We included only clinically determined TNBC cases in the classification. TNBCtype has a pre-filter step where it reports samples that the algorithm does not consider as TNBC and then stops. In a first run, such samples were identified and then removed from the TNBC data set. The filtered data set was again passed to TNBCtype. Prior to the classification TNBC data was mean-centered.
6. *HDPP*. For classification we used the centroids reported by Staaf et al. [6], using Pearson correlation. Prior to the classification data was mean-centered and matched to centroids based on gene identifier. We used the highest centroid correlation >0 to assign a class for each sample.
7. *SDPP*. For classification we used a *genefu* R package [7] version 2.10.0 implementation of the original signature [8]. Prior to the classification data was mean centered across all samples.

8. *SCMOD2*. For classification we used a *genefu* R package [7] version 2.10.0 implementation of the original signature. Prior to the classification data was mean centered across all samples.
9. *GGI*. For classification we used a *genefu* R package [7] version 2.10.0 implementation of the original signature. Prior to the classification data was mean centered across all samples. The genefu ggi() function requires grade to be supplied, and we used the Nottingham grade index from registry data for this purpose.
10. *Gene70*. For classification we used a *genefu* R package [7] version 2.10.0 implementation of the original signature. Prior to the classification data was mean centered across all samples. We used the scale option for the classification in the genfur gene70() function.
11. *Oncotype DX*. For classification we used a *genefu* R package [7] version 2.10.0 implementation of the original signature. Prior to the classification data was mean centered across all samples.
12. *Gene76*. For classification we used a *genefu* R package [7] version 2.10.0 implementation of the original signature. Prior to the classification data was mean centered across all samples. ER status from registry data was supplied as needed for the function.
13. *Endopredict*. For classification we used a *genefu* R package [7] version 2.10.0 implementation of the original signature. Prior to the classification data was mean centered across all samples.
14. *Genius*. For classification we used a *genefu* R package [7] version 2.10.0 implementation of the original signature. Prior to the classification data was mean centered across all samples.
15. *ROR-S*. We used centroid correlations from PAM50 above and the equation from [4] to calculate a ROR-score. All samples were divided into three equally sized groups, low-risk, medium/intermediate-risk, and high-risk based on quantiles of the ROR-score.
16. *ROR-P*. We used centroid correlations from PAM50 above and the equation from [4, 9] to calculate a ROR-score. All samples were divided into three equally sized groups, low-risk, medium/intermediate-risk, and high-risk based on quantiles of the ROR-score. The proliferation signature score used in the equation was calculated based on the gene list from Nielsen et al. [9] using mean centered data, as outlined in that study.
17. *ROR-T*. We used centroid correlations from PAM50 above and the equation from [4, 9] to calculate a ROR-score. All samples were divided into three equally sized groups, low-risk, medium/intermediate-risk, and high-risk based on quantiles of the ROR-score.
18. *ROR-PT*. We used centroid correlations from PAM50 above and the equations from [4] to calculate a ROR-score. Samples were divided into three groups, low-risk, medium/intermediate-risk, and high-risk based on quantiles of the ROR-score. The proliferation signature scored was calculated based on the gene list from Nielsen et al. [9] using mean centered data as outlined in that study.
19. *ROR-Tot*. Equation, optimized weights and cut-offs for classification were obtained from [10]. The proliferation signature score was calculated based on the gene list from [10] using mean centered data as outlined in that study.

References

1. Saal LH, Vallon-Christersson J, Hakkinen J, Hegardt C, Grabau D, Winter C, et al: **The Sweden Cancerome Analysis Network - Breast (SCAN-B) Initiative: a large-**

scale multicenter infrastructure towards implementation of breast cancer genomic analyses in the clinical routine. *Genome Med* 2015, **7:**20.

2. Ryden L, Loman N, Larsson C, Hegardt C, Vallon-Christersson J, Malmberg M, et al: **Minimizing inequality in access to precision medicine in breast cancer by real-time population-based molecular analysis in the SCAN-B initiative.** *Br J Surg* 2018, **105:**e158-e168.
3. Brueffer C, Vallon-Christersson J, Grabau† D, Ehinger A, Häkkinen J, Hegardt C, et al: **Clinical Value of RNA Sequencing–Based Classifiers for Prediction of the Five Conventional Breast Cancer Biomarkers: A Report From the Population-Based Multicenter Sweden Cancerome Analysis Network—Breast Initiative.** *JCO Precision Oncology* 2018**:**1-18.
4. Parker JS, Mullins M, Cheang MC, Leung S, Voduc D, Vickery T, et al: **Supervised risk predictor of breast cancer based on intrinsic subtypes.** *J Clin Oncol* 2009, **27:**1160-1167.
5. Chen X, Li J, Gray WH, Lehmann BD, Bauer JA, Shyr Y, et al: **TNBCtype: A Subtyping Tool for Triple-Negative Breast Cancer.** *Cancer Inform* 2012, **11:**147-156.
6. Staaf J, Ringner M, Vallon-Christersson J, Jonsson G, Bendahl PO, Holm K, et al: **Identification of subtypes in human epidermal growth factor receptor 2--positive breast cancer reveals a gene signature prognostic of outcome.** *J Clin Oncol* 2010, **28:**1813-1820.
7. Gendoo DM, Ratanasirigulchai N, Schroder MS, Pare L, Parker JS, Prat A, et al: **Genefu: an R/Bioconductor package for computation of gene expression-based signatures in breast cancer.** *Bioinformatics* 2016, **32:**1097-1099.
8. Finak G, Bertos N, Pepin F, Sadekova S, Souleimanova M, Zhao H, et al: **Stromal gene expression predicts clinical outcome in breast cancer.** *Nat Med* 2008, **14:**518-527.
9. Nielsen TO, Parker JS, Leung S, Voduc D, Ebbert M, Vickery T, et al: **A comparison of PAM50 intrinsic subtyping with immunohistochemistry and clinical prognostic factors in tamoxifen-treated estrogen receptor-positive breast cancer.** *Clin Cancer Res* 2010, **16:**5222-5232.
10. Filipits M, Nielsen TO, Rudas M, Greil R, Stoger H, Jakesz R, et al: **The PAM50 risk-of-recurrence score predicts risk for late distant recurrence after endocrine therapy in postmenopausal women with endocrine-responsive early breast cancer.** *Clin Cancer Res* 2014, **20:**1298-1305.

Supplementary Figure S1

Univariate analysis of the association with overall survival for 19 gene signatures stratified into nine clinical assessment groups. Signature classes <8% in size were excluded from analyses in respective assessment group. (**A**) ER+/HER2-/LN- with endocrine (Endo) therapy only. (**B**) ER+/HER2-/LN- with adjuvant chemotherapy (ACT) and endocrine therapy. (**C**) ER+/HER2-/LN- disease with no adjuvant treatment. (**D**) ER+/HER2-/LN+ with endocrine therapy only. (**E**) ER+/HER2-/LN- with adjuvant chemotherapy (ACT) and endocrine therapy. (**F**) HER2+/ER- disease with anti-HER2 (mAB) and adjuvant chemotherapy treatment. (**G**) HER2+/ER+ disease with anti-HER2 (mAB), adjuvant chemotherapy, and endocrine treatment. (**H**) TNBC with adjuvant therapy. (**I**) TNBC with no adjuvant therapy indicated.

A)

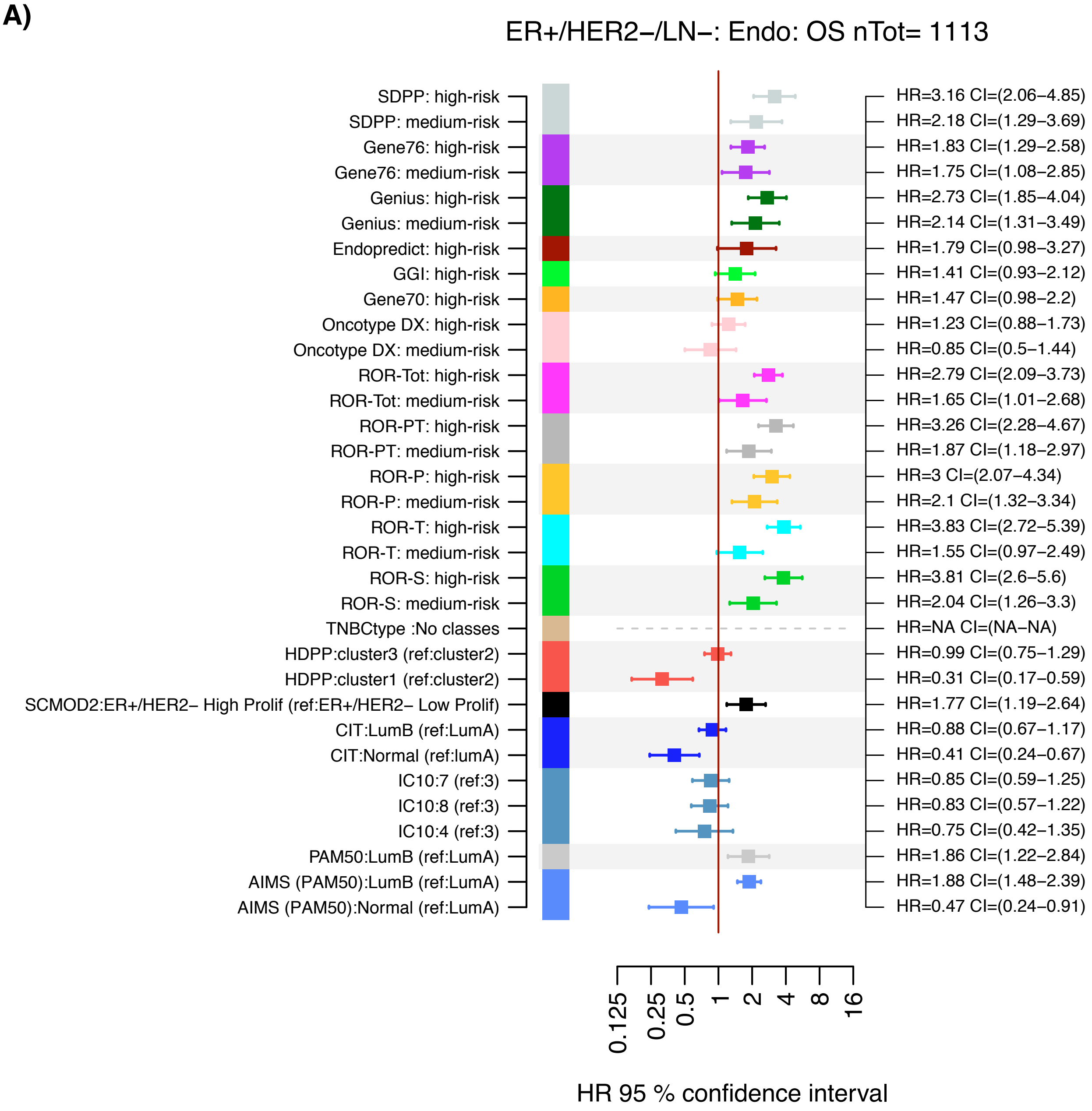

- If not stated otherwise (e.g. ref:XXX), reference is the low-risk group
- No classes: no classes available
- No event: at least one class does not harbor any events, meaning that the analysis fails.
- Only one class: only one class for the signature available, meaning no analysis

B)

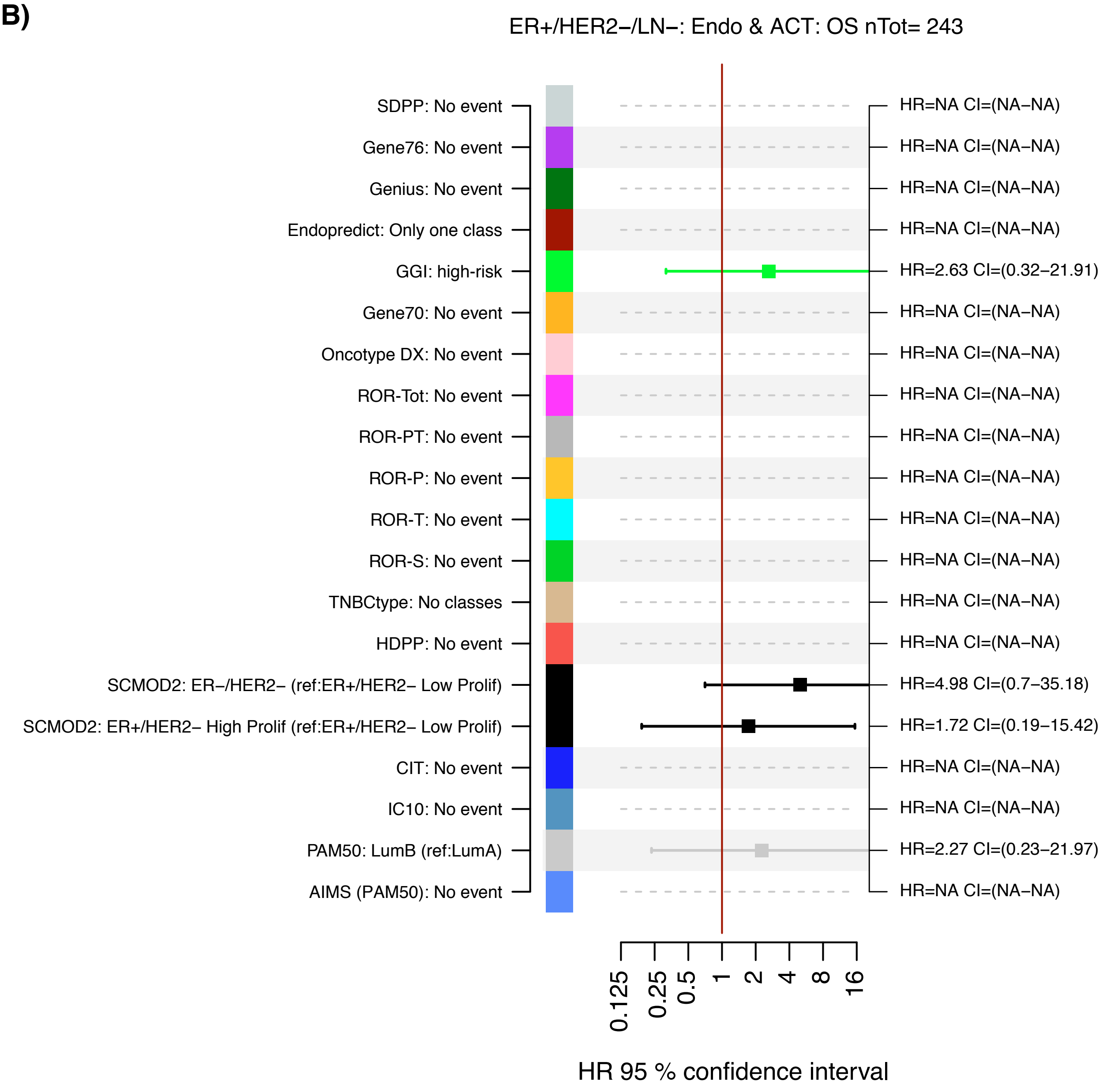

- If not stated otherwise (e.g. ref:XXX), reference is the low-risk group
- No classes: no classes available
- No event: at least one class does not harbor any events, meaning that the analysis fails.
- Only one class: only one class for the signature available, meaning no analysis

C)

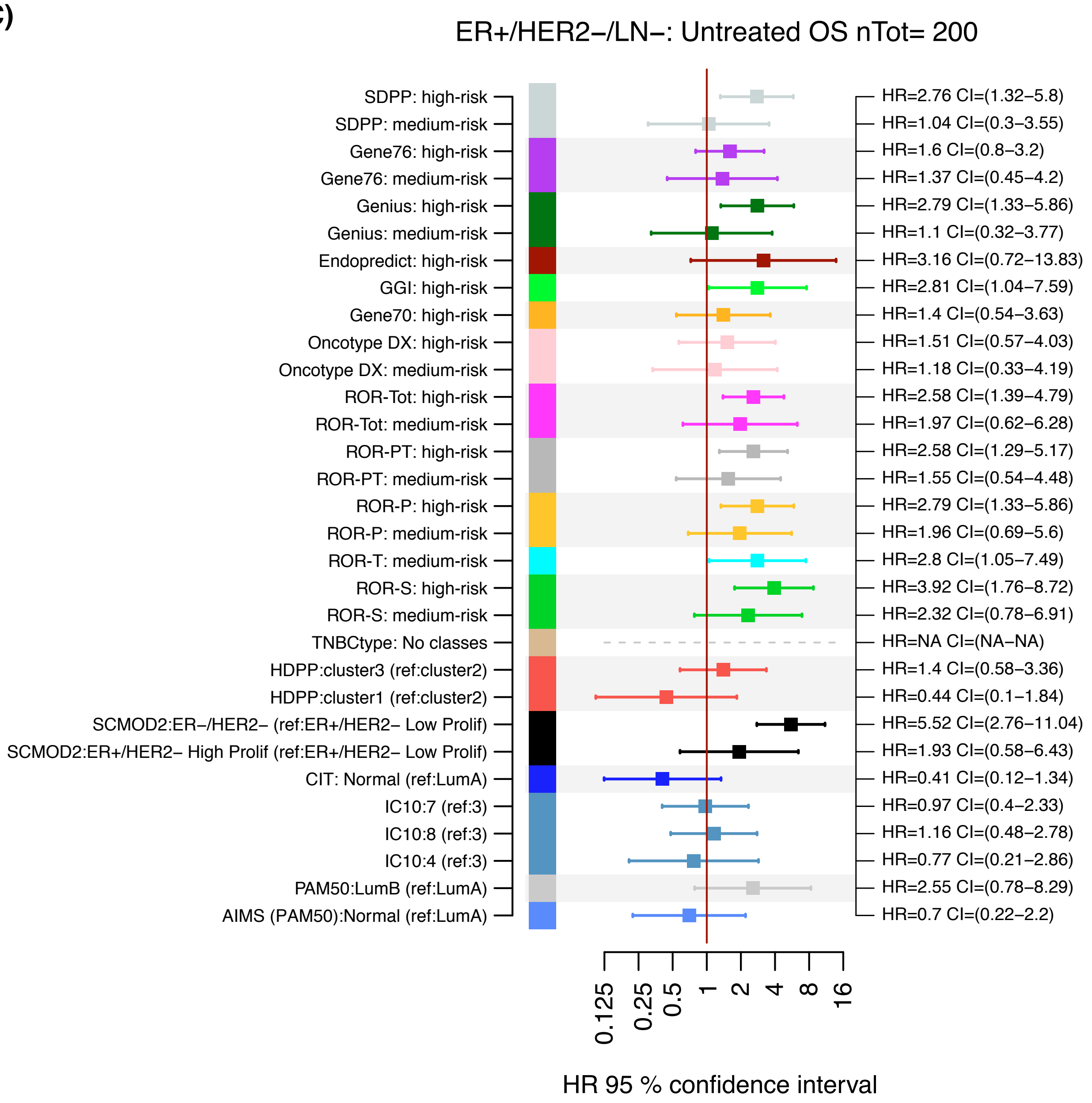

- If not stated otherwise (e.g. ref:XXX), reference is the low-risk group
- No classes: no classes available
- No event: at least one class does not harbor any events, meaning that the analysis fails.
- Only one class: only one class for the signature available, meaning no analysis

D)

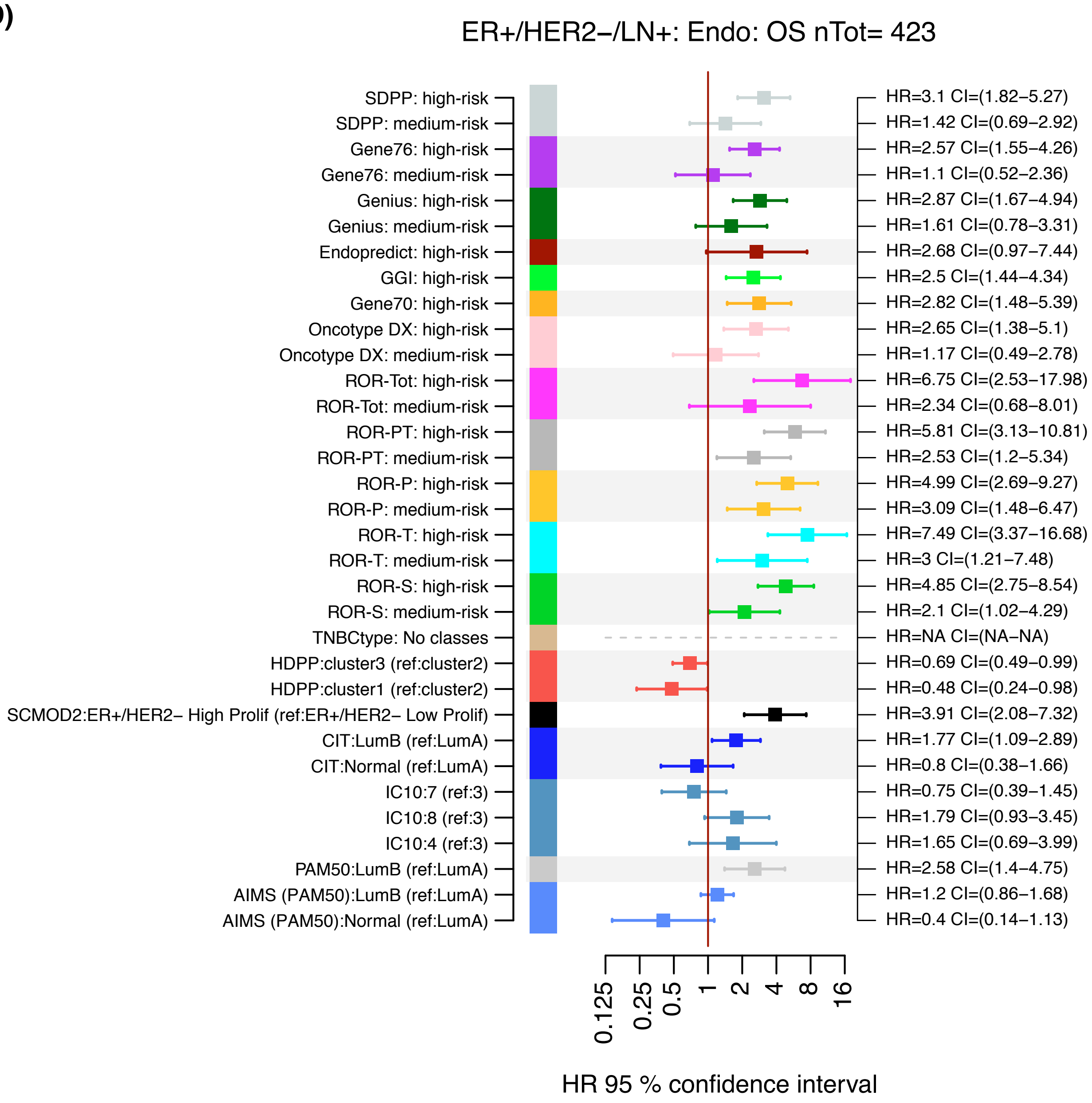

- If not stated otherwise (e.g. ref:XXX), reference is the low-risk group
- No classes: no classes available
- No event: at least one class does not harbor any events, meaning that the analysis fails.
- Only one class: only one class for the signature available, meaning no analysis

E)

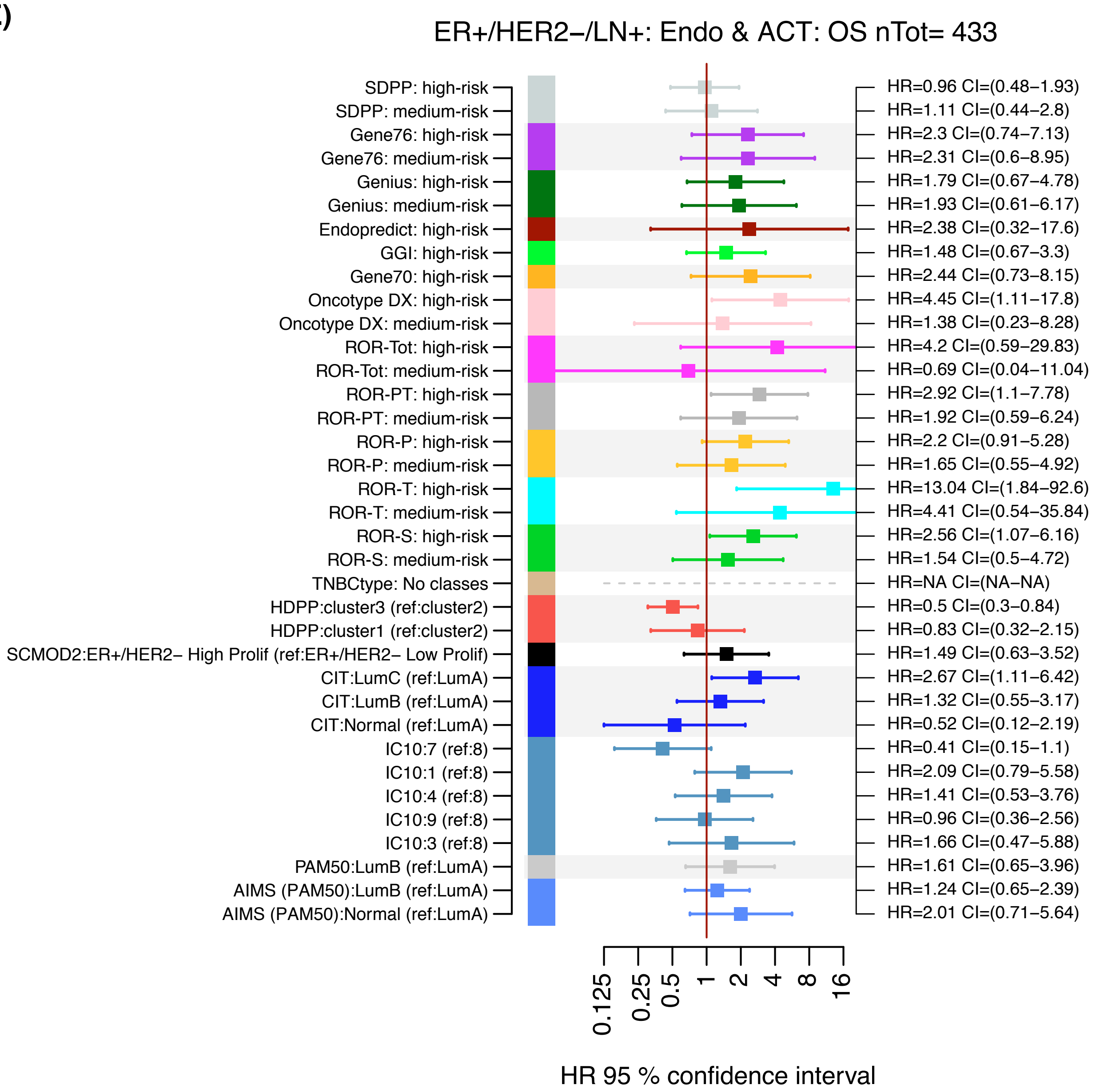

- If not stated otherwise (e.g. ref:XXX), reference is the low-risk group
- No classes: no classes available
- No event: at least one class does not harbor any events, meaning that the analysis fails.
- Only one class: only one class for the signature available, meaning no analysis

F)

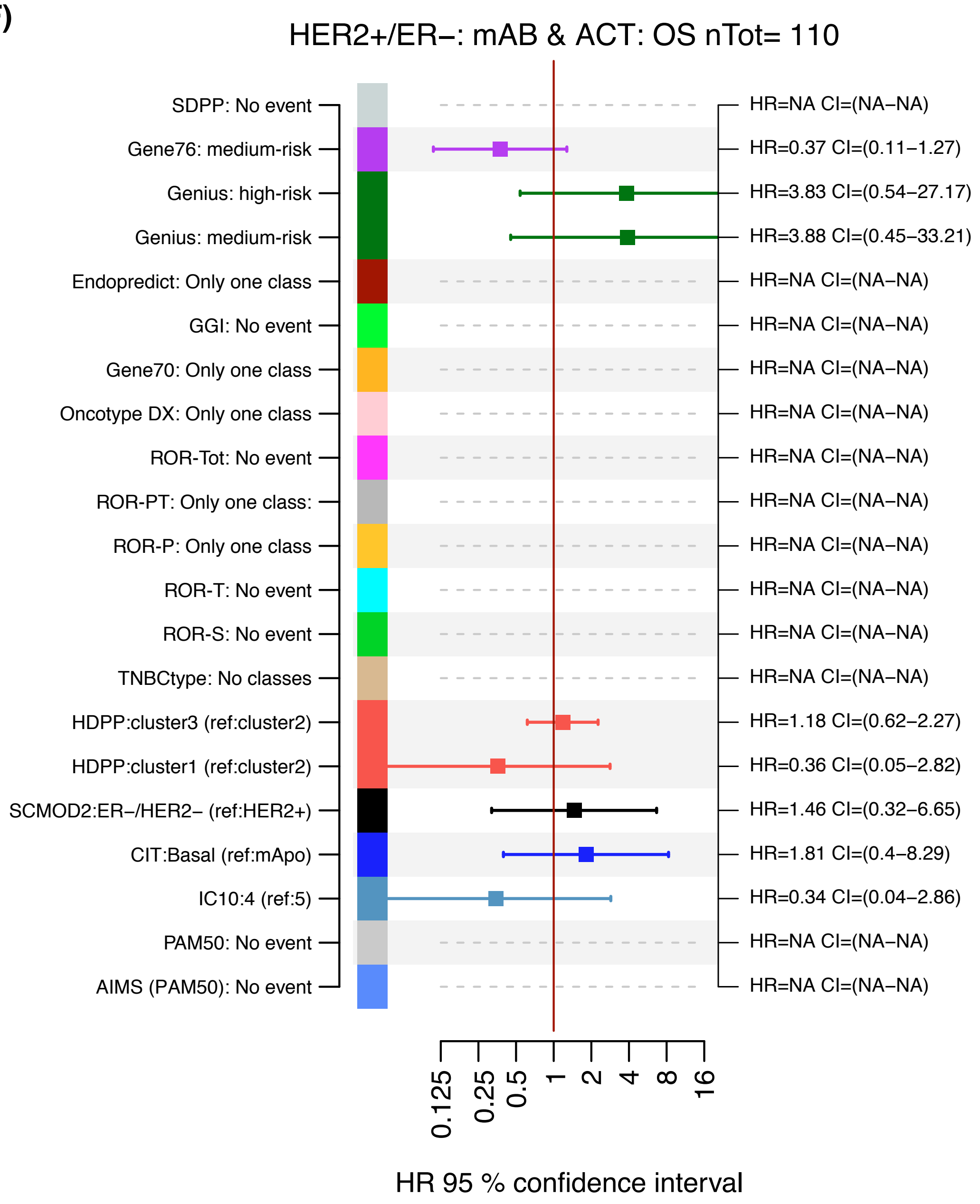

- If not stated otherwise (e.g. ref:XXX), reference is the low-risk group
- No classes: no classes available
- No event: at least one class does not harbor any events, meaning that the analysis fails.
- Only one class: only one class for the signature available, meaning no analysis

G)

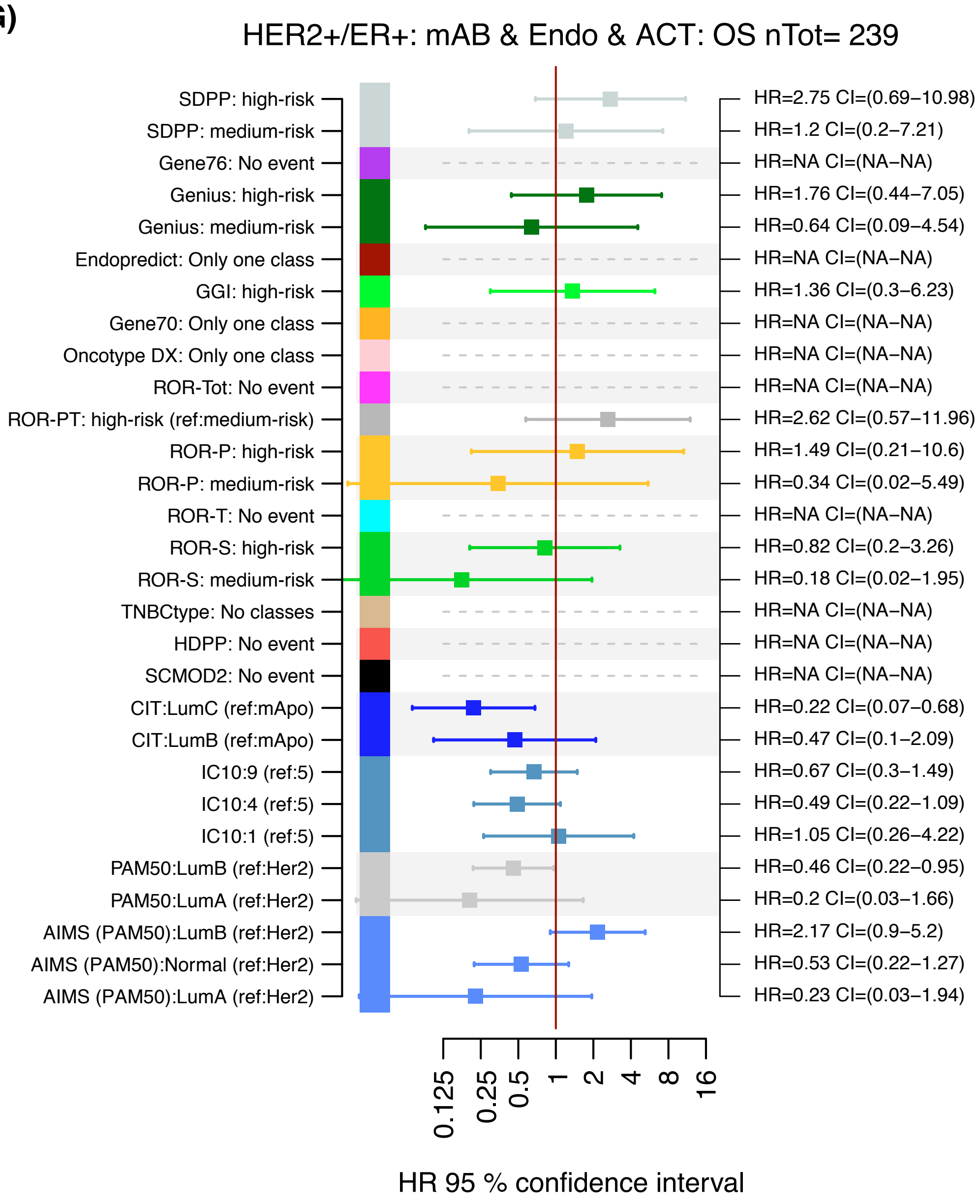

- If not stated otherwise (e.g. ref:XXX), reference is the low-risk group
- No classes: no classes available
- No event: at least one class does not harbor any events, meaning that the analysis fails.
- Only one class: only one class for the signature available, meaning no analysis

H)

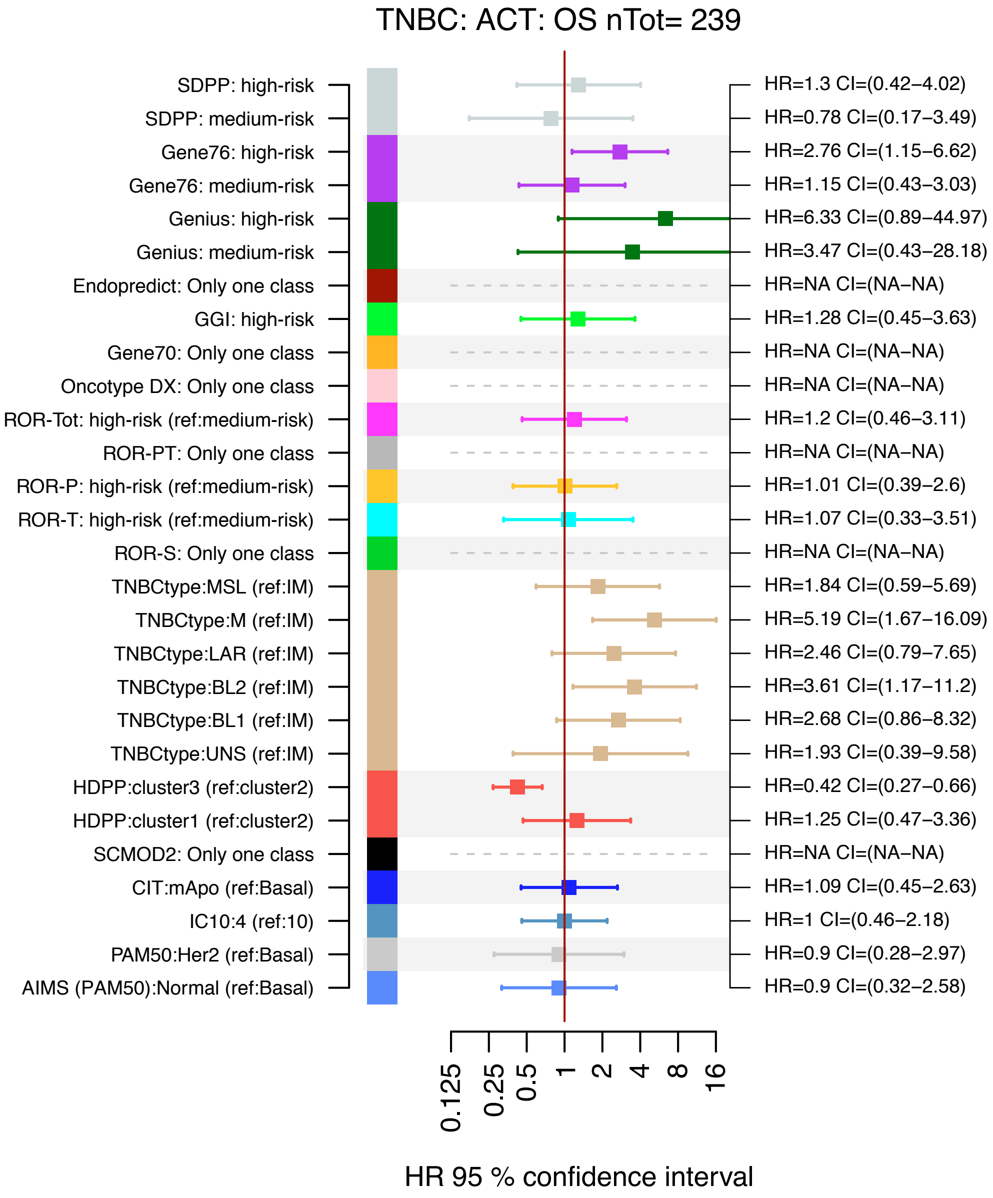

- If not stated otherwise (e.g. ref:XXX), reference is the low-risk group
- No classes: no classes available
- No event: at least one class does not harbor any events, meaning that the analysis fails.
- Only one class: only one class for the signature available, meaning no analysis

I)

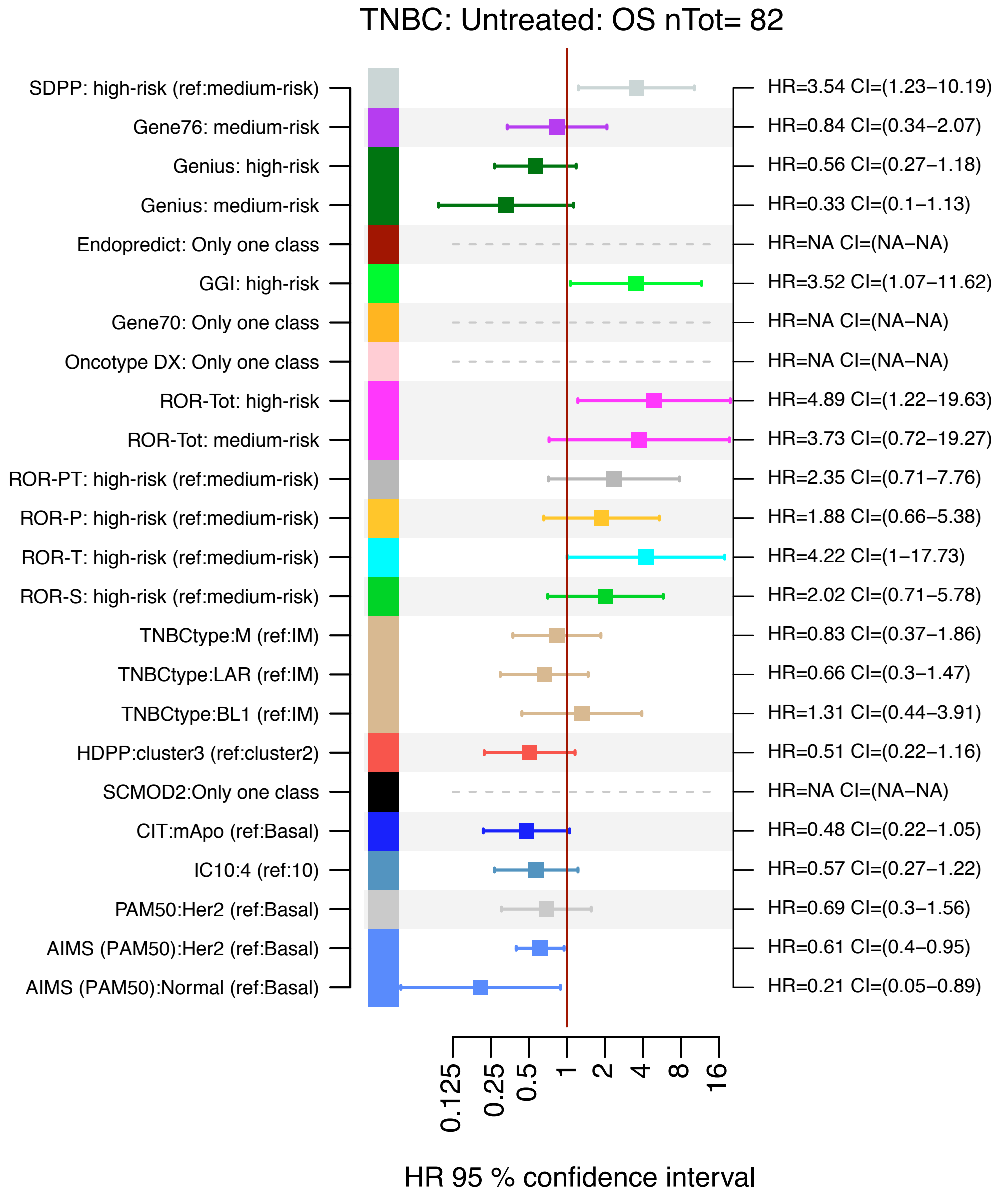

- If not stated otherwise (e.g. ref:XXX), reference is the low-risk group
- No classes: no classes available
- No event: at least one class does not harbor any events, meaning that the analysis fails.
- Only one class: only one class for the signature available, meaning no analysis

Supplementary Figure S2

Multivariable analysis of the association with overall survival for 19 gene signatures stratified into nine clinical assessment groups. Signature classes <8% in size were excluded from analyses in respective assessment group. Right hand side axis in forest plots indicates group size and hazard ratio with 95% confidence interval. (**A**) ER+/HER2-/LN- with endocrine therapy only. (**B**) ER+/HER2-/LN- with adjuvant chemotherapy (ACT) and endocrine therapy. (**C**) ER+/HER2-/LN- disease with no adjuvant treatment. (**D**) ER+/HER2-/LN+ with endocrine therapy only. (**E**) ER+/HER2-/LN- with adjuvant chemotherapy (ACT) and endocrine therapy. (**F**) HER2+/ER- disease with anti-HER2 (mAB) and adjuvant chemotherapy treatment. (**G**) HER2+/ER+ disease with anti-HER2 (mAB), adjuvant chemotherapy, and endocrine treatment. (**H**) TNBC with adjuvant therapy. (**I**) TNBC with no adjuvant therapy indicated.

A)

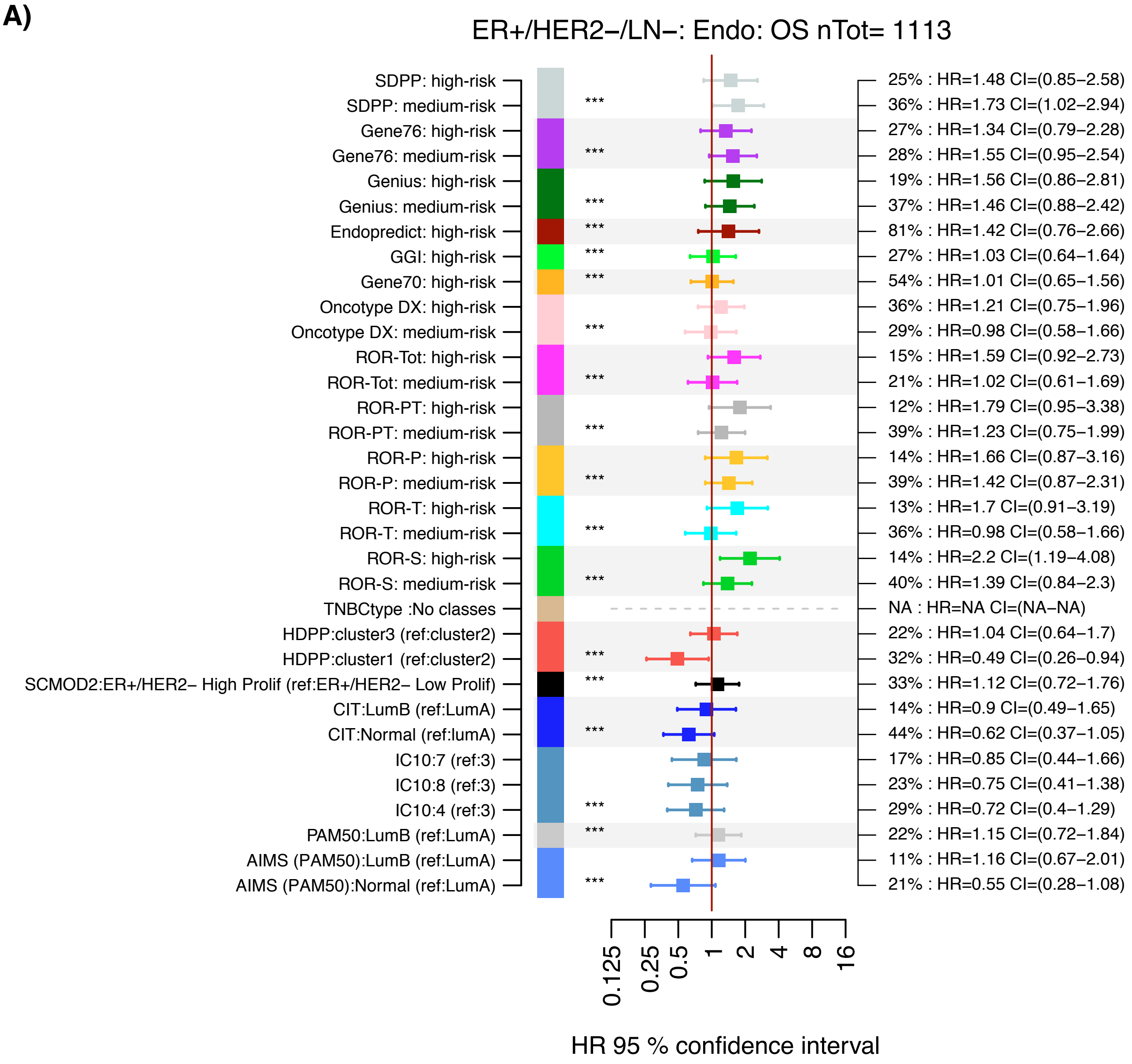

- Covariates: tumor size (mm), patient age, lymph node status (when applicable, N+ or N0), tumor grade (1,2,3)
- IF not stated otherwise (e.g. ref:XXX), reference is the low-risk group
- No classes: no classes available
- No event: at least one class does not harbor any events, meaning that the analysis fails.
- Only one class: only one class for the signature available, meaning no analysis

*,**,*** indicates significance levels (p<0.05, p<0.01, p<0.001) of likelihood ratio test

B)

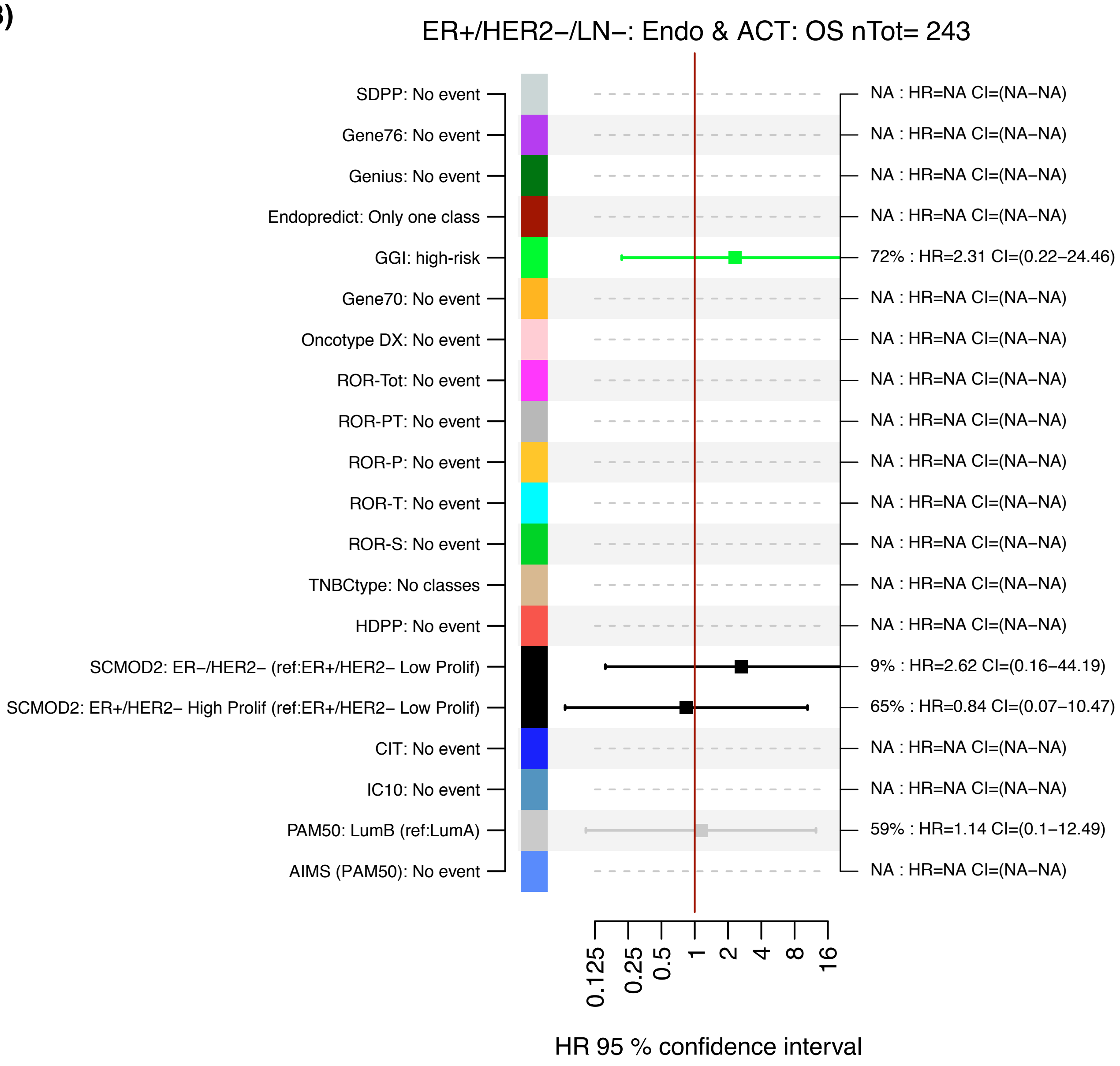

- Covariates: tumor size (mm), patient age, lymph node status (when applicable, N+ or N0), tumor grade (1,2,3)
- IF not stated otherwise (e.g. ref:XXX), reference is the low-risk group
- No classes: no classes available
- No event: at least one class does not harbor any events, meaning that the analysis fails.
- Only one class: only one class for the signature available, meaning no analysis

*,**,*** indicates significance levels (p<0.05, p<0.01, p<0.001) of likelihood ratio test

C)

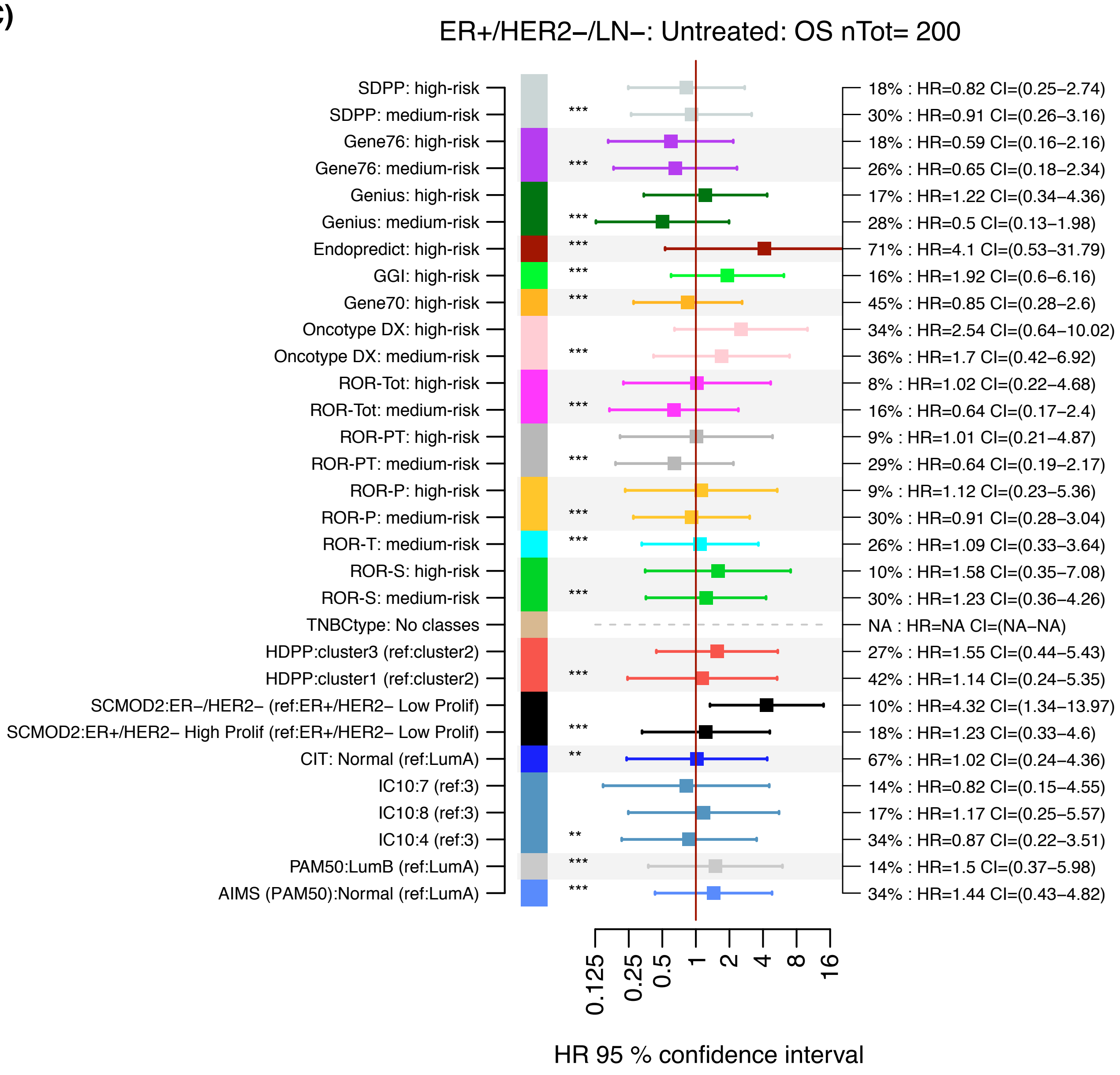

- Covariates: tumor size (mm), patient age, lymph node status (when applicable, N+ or N0), tumor grade (1,2,3)
- IF not stated otherwise (e.g. ref:XXX), reference is the low-risk group
- No classes: no classes available
- No event: at least one class does not harbor any events, meaning that the analysis fails.
- Only one class: only one class for the signature available, meaning no analysis

*,**,*** indicates significance levels (p<0.05, p<0.01, p<0.001) of likelihood ratio test

D)

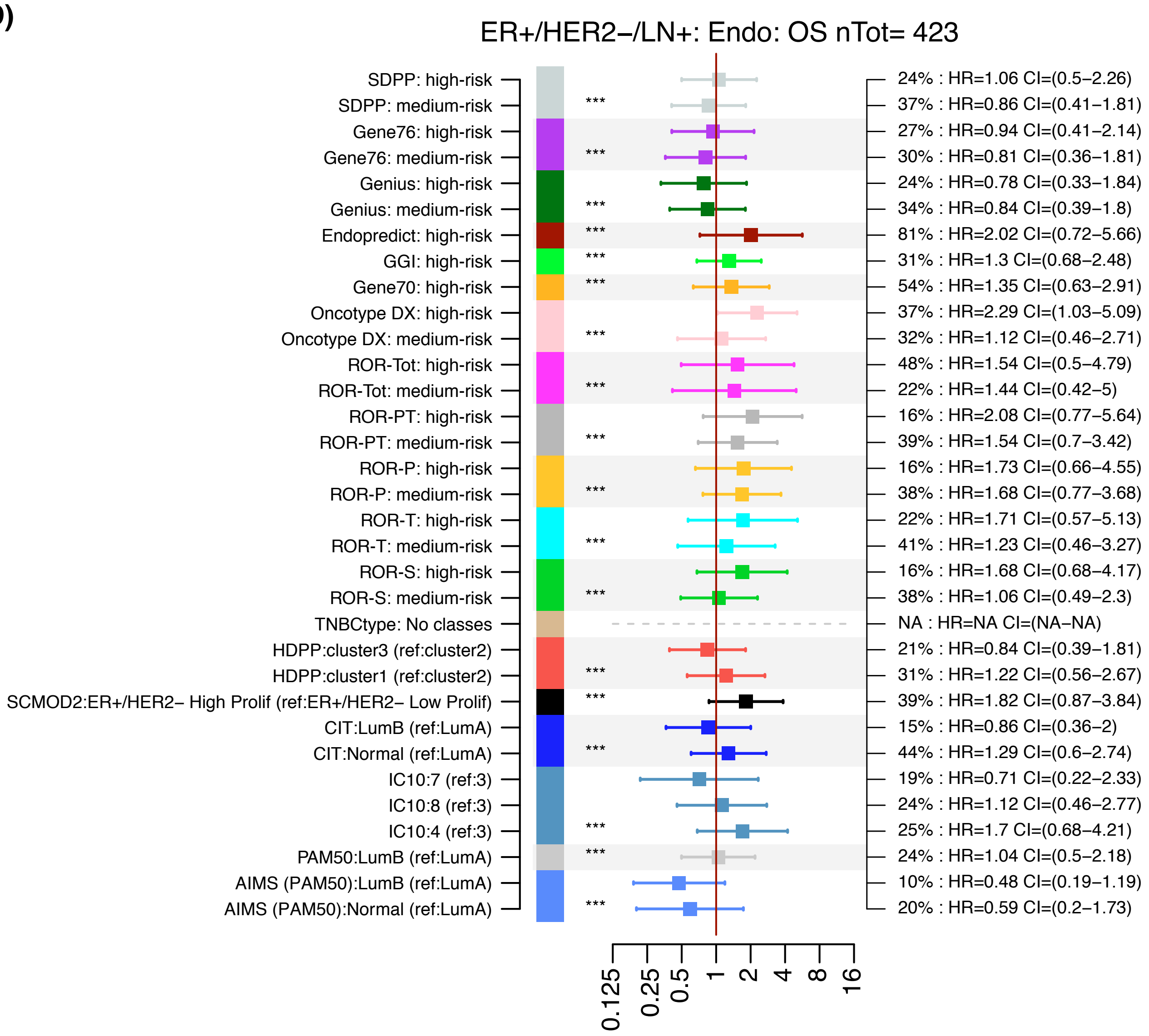

- Covariates: tumor size (mm), patient age, lymph node status (when applicable, N+ or N0), tumor grade (1,2,3)
- IF not stated otherwise (e.g. ref:XXX), reference is the low-risk group
- No classes: no classes available
- No event: at least one class does not harbor any events, meaning that the analysis fails.
- Only one class: only one class for the signature available, meaning no analysis

*,**,*** indicates significance levels (p<0.05, p<0.01, p<0.001) of likelihood ratio test

E)

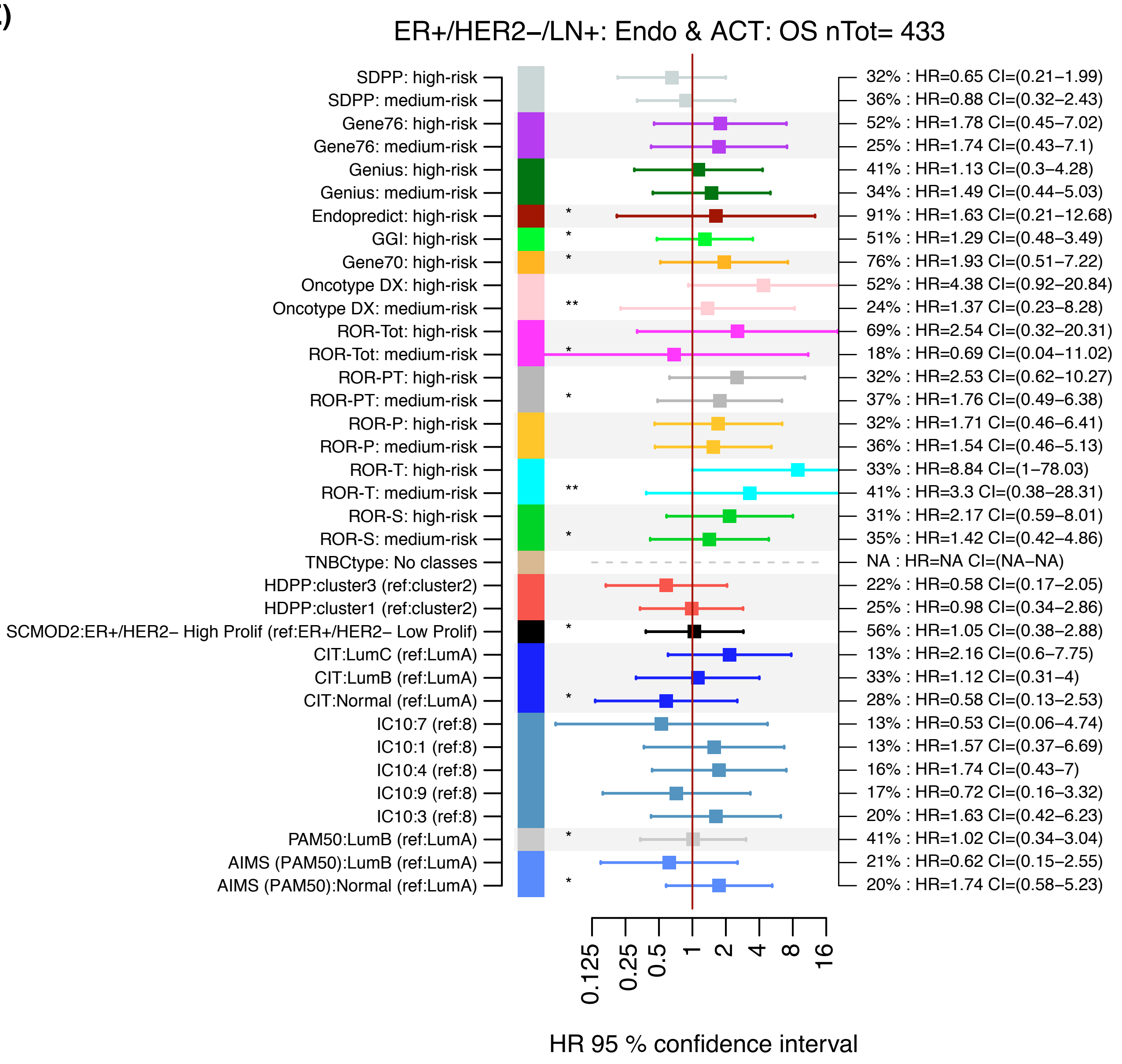

- Covariates: tumor size (mm), patient age, lymph node status (when applicable, N+ or N0), tumor grade (1,2,3)
- IF not stated otherwise (e.g. ref:XXX), reference is the low-risk group
- No classes: no classes available
- No event: at least one class does not harbor any events, meaning that the analysis fails.
- Only one class: only one class for the signature available, meaning no analysis

*,**,*** indicates significance levels (p<0.05, p<0.01, p<0.001) of likelihood ratio test

F)

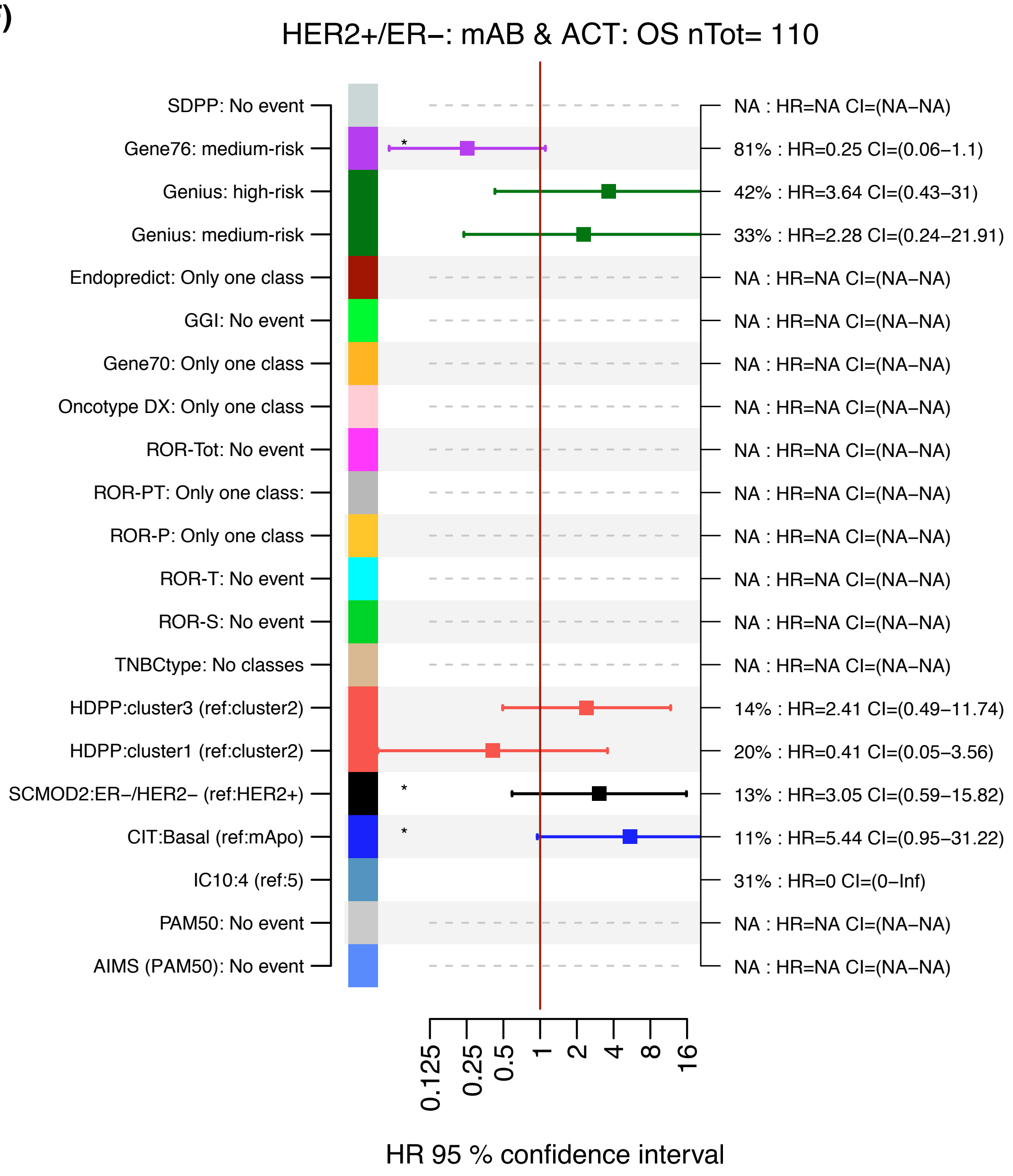

- Covariates: tumor size (mm), patient age, lymph node status (when applicable, N+ or N0), tumor grade (1,2,3)
- IF not stated otherwise (e.g. ref:XXX), reference is the low-risk group
- No classes: no classes available
- No event: at least one class does not harbor any events, meaning that the analysis fails.
- Only one class: only one class for the signature available, meaning no analysis

*,**,*** indicates significance levels (p<0.05, p<0.01, p<0.001) of likelihood ratio test

G)

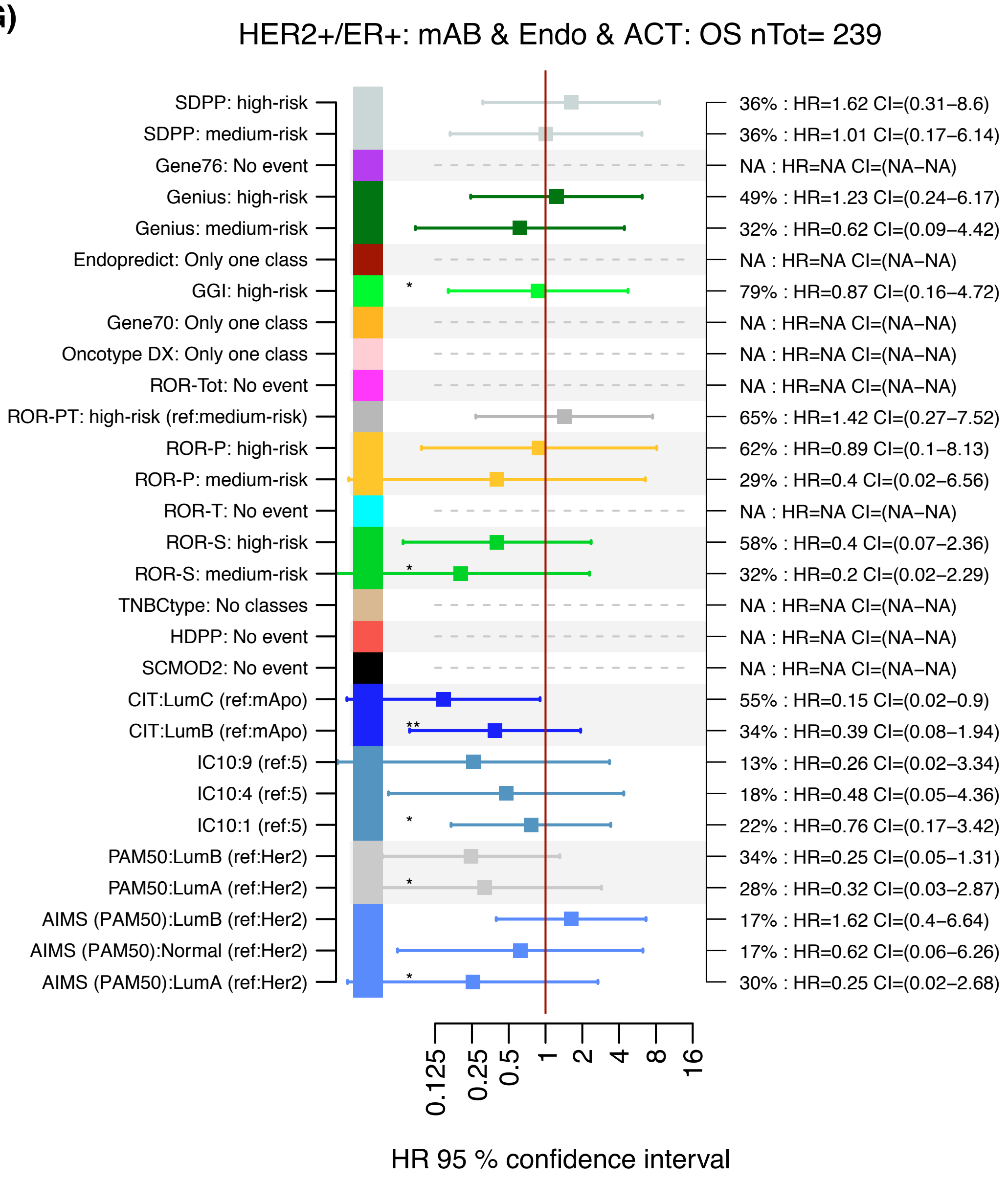

- Covariates: tumor size (mm), patient age, lymph node status (when applicable, N+ or N0), tumor grade (1,2,3)
- IF not stated otherwise (e.g. ref:XXX), reference is the low-risk group
- No classes: no classes available
- No event: at least one class does not harbor any events, meaning that the analysis fails.
- Only one class: only one class for the signature available, meaning no analysis

*,**,*** indicates significance levels (p<0.05, p<0.01, p<0.001) of likelihood ratio test

H)

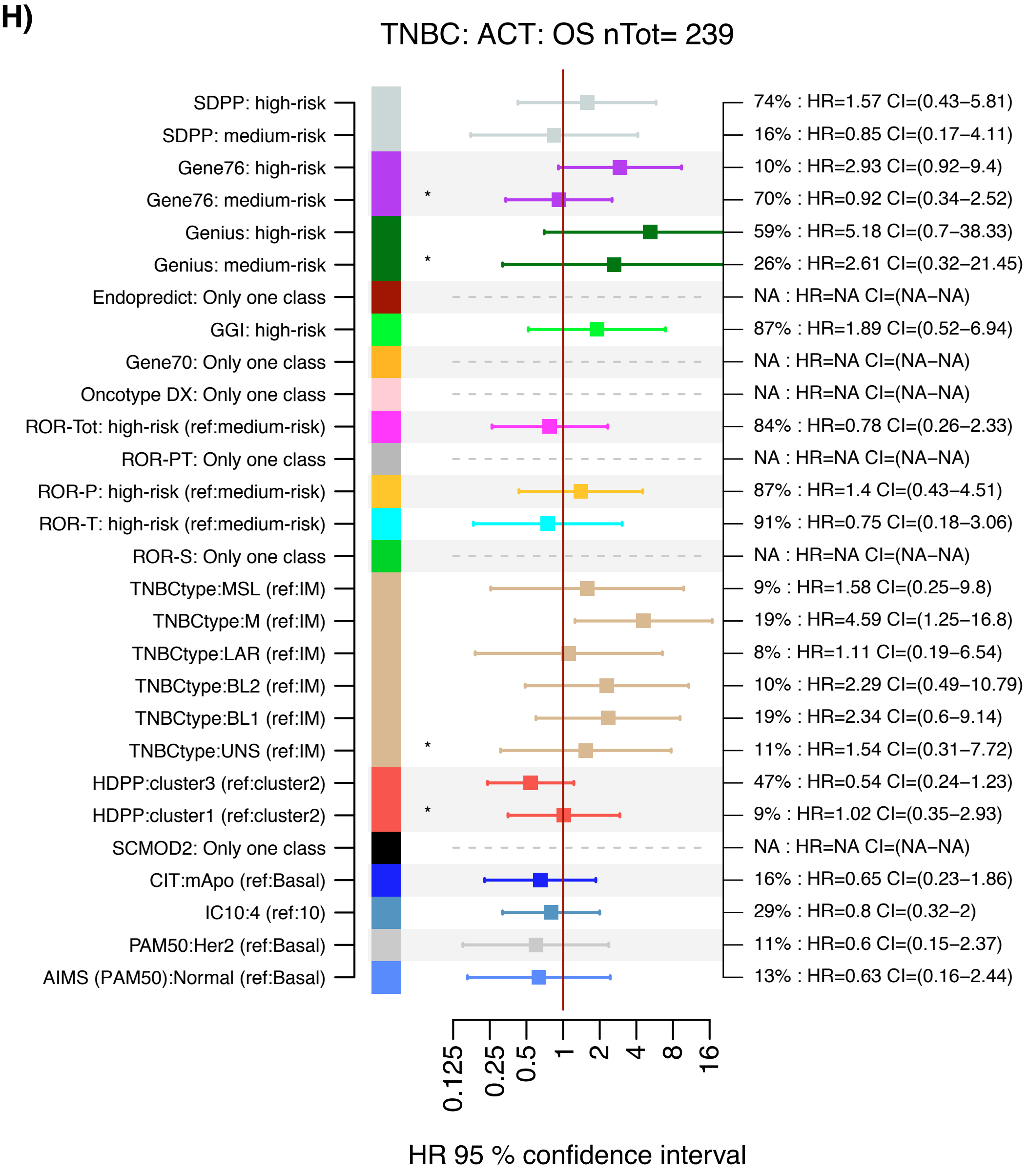

- Covariates: tumor size (mm), patient age, lymph node status (when applicable, N+ or N0), tumor grade (1,2,3)
- IF not stated otherwise (e.g. ref:XXX), reference is the low-risk group
- No classes: no classes available
- No event: at least one class does not harbor any events, meaning that the analysis fails.
- Only one class: only one class for the signature available, meaning no analysis

*,**,*** indicates significance levels (p<0.05, p<0.01, p<0.001) of likelihood ratio test

I)

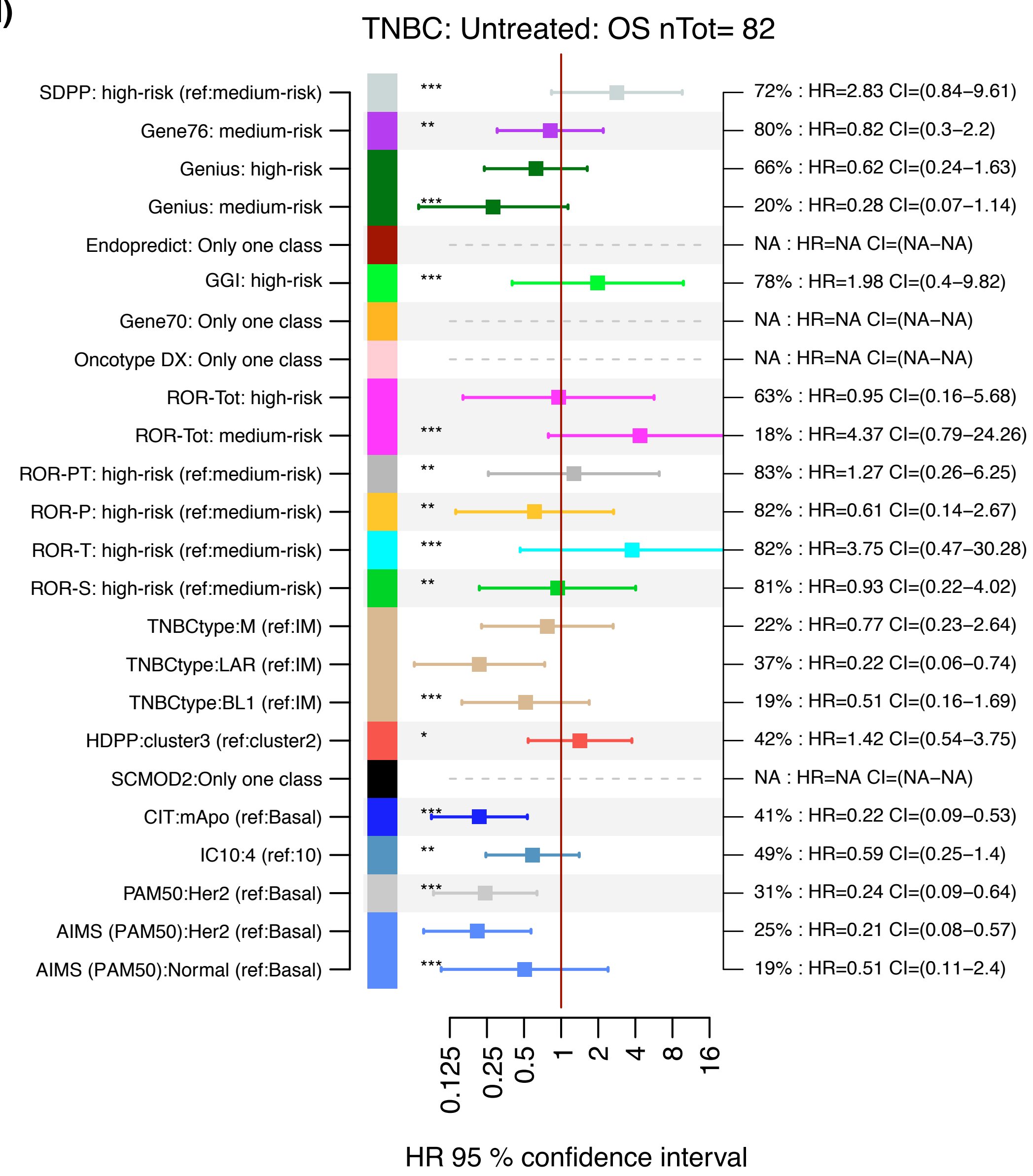

- Covariates: tumor size (mm), patient age, lymph node status (when applicable, N+ or N0), tumor grade (1,2,3)
- IF not stated otherwise (e.g. ref:XXX), reference is the low-risk group
- No classes: no classes available
- No event: at least one class does not harbor any events, meaning that the analysis fails.
- Only one class: only one class for the signature available, meaning no analysis

*,**,*** indicates significance levels (p<0.05, p<0.01, p<0.001) of likelihood ratio test

Supplementary Figure S3. Classification consensus in clinical assessment groups for risk prediction signatures.

For each panel, two heatmaps are shown. Left: Percentage of exact risk class agreement (i.e. % of cases with the same predictions) for risk prediction signature pairs in a clinical assessment group using all available signature classes in the individual comparisons. Calculated from the diagonal of e.g. a 2x2 or 3x3 table. Right: Similar display as in left, both now for only low-risk and high-risk classified samples. In this analysis, all patients with an intermediate-risk prediction in a signature pair comparison were omitted before calculation of the exact agreement. (**A**) ER+/HER2-/LN- with endocrine treatment. (**B**) ER+/HER2-/LN- with ACT & endocrine treatment. (**C**) ER+/HER2-/LN- untreated. (**D**) ER+/HER2-/LN+ with endocrine treatment. (**E**) ER+/HER2-/LN+ with ACT & endocrine treatment. (**F**) HER2+/ER- with anti-HER2 blockade and ACT. (**G**) HER2+/ER+ with anti-HER2 blockade, ACT and endocrine treatment. (**H**) TNBC with ACT. (**I**) TNBC untreated.

Panel legends: Endo: endocrine therapy. ACT: adjuvant chemotherapy. Immune: anti-HER2 treatment.

A)

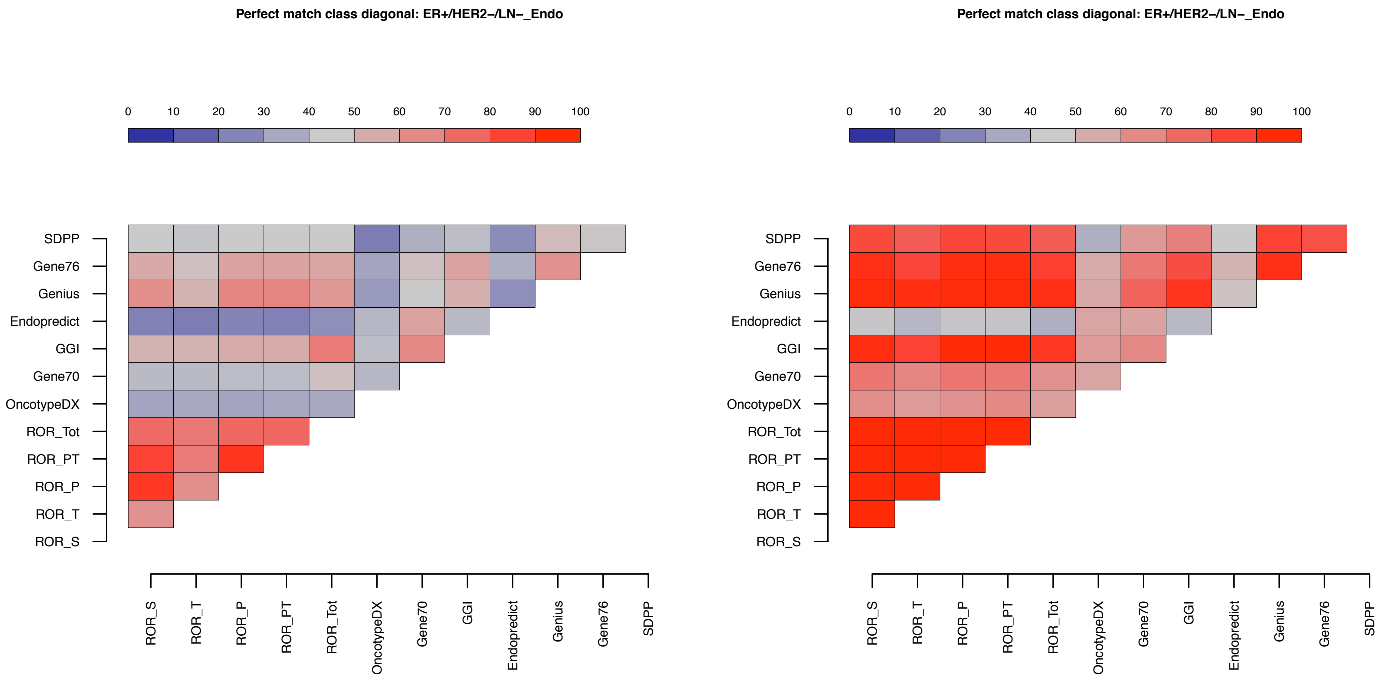

B)

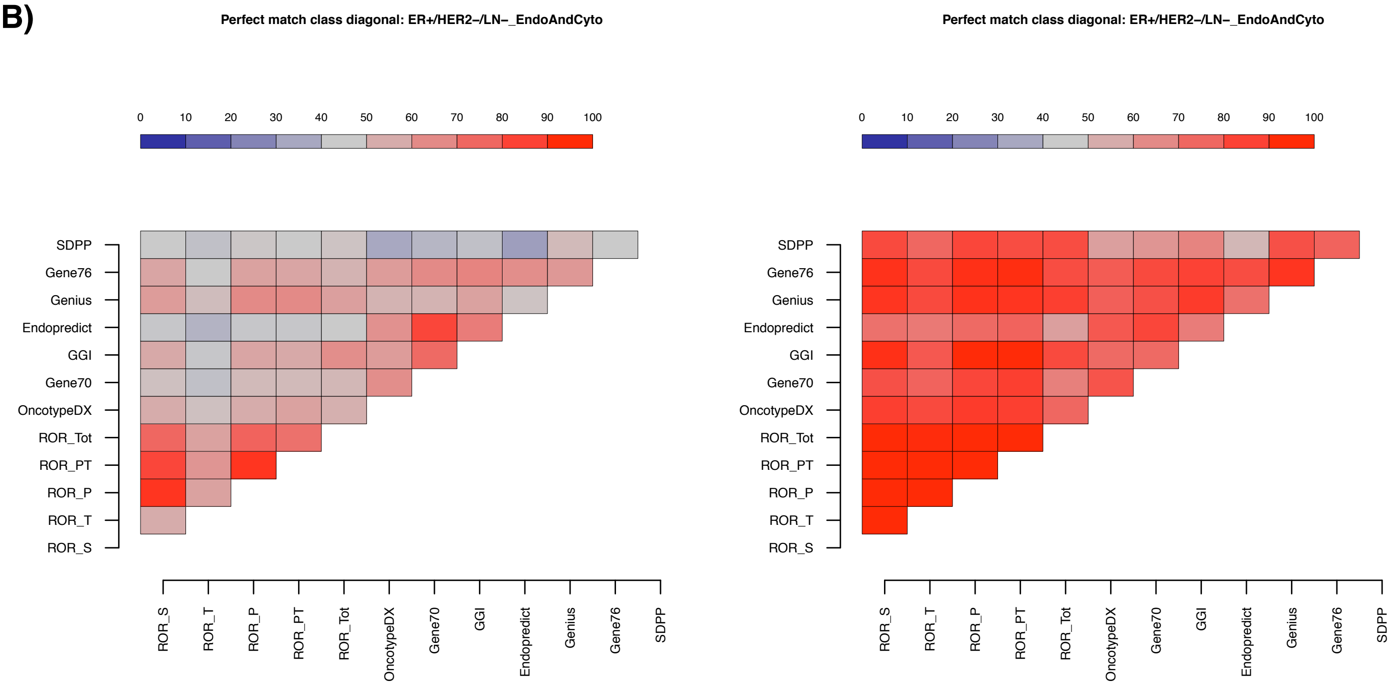

C)

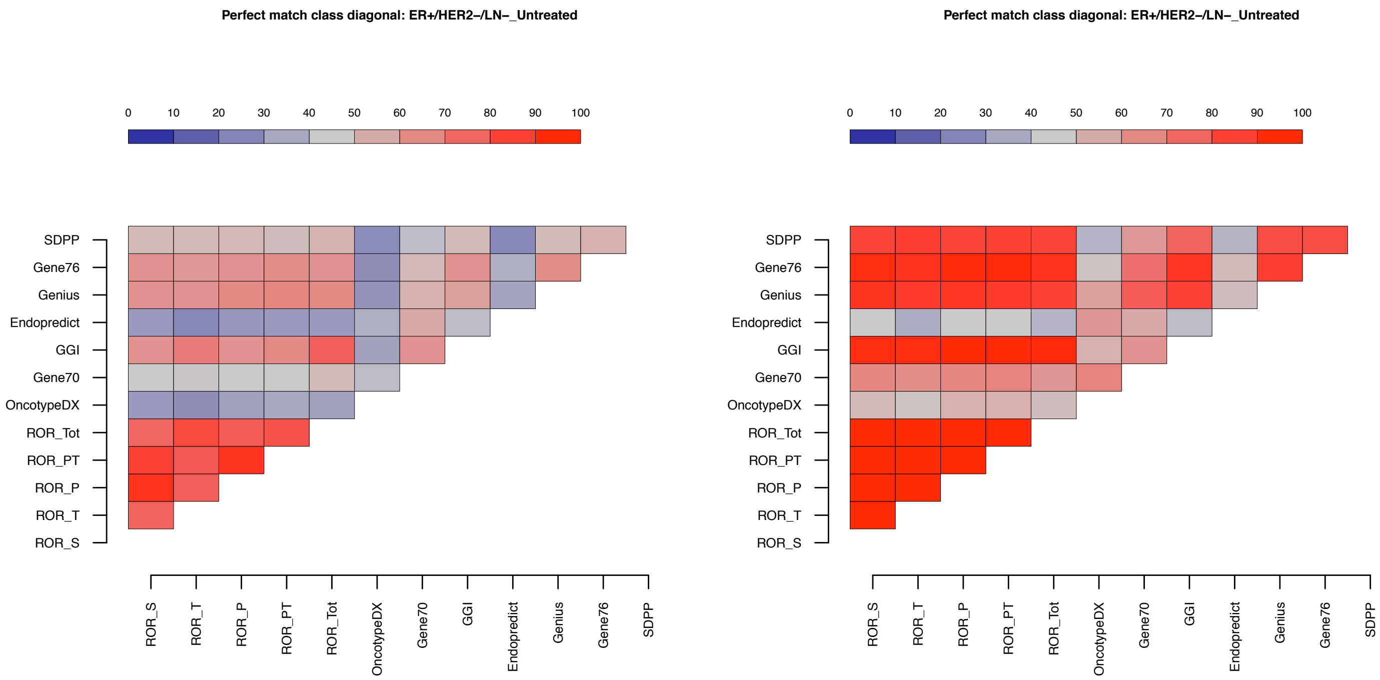

Perfect match class diagonal: ER+/HER2−/LN−_Untreated
0 10 20 30 40 50 60 70 80 90 100
SDPP
Gene76
Genius
Endopredict
GGI
Gene70
OncotypeDX
ROR_Tot
ROR_PT
ROR_P
ROR_T
ROR_S
Perfect match class diagonal: ER+/HER2−/LN−_Untreated

D)

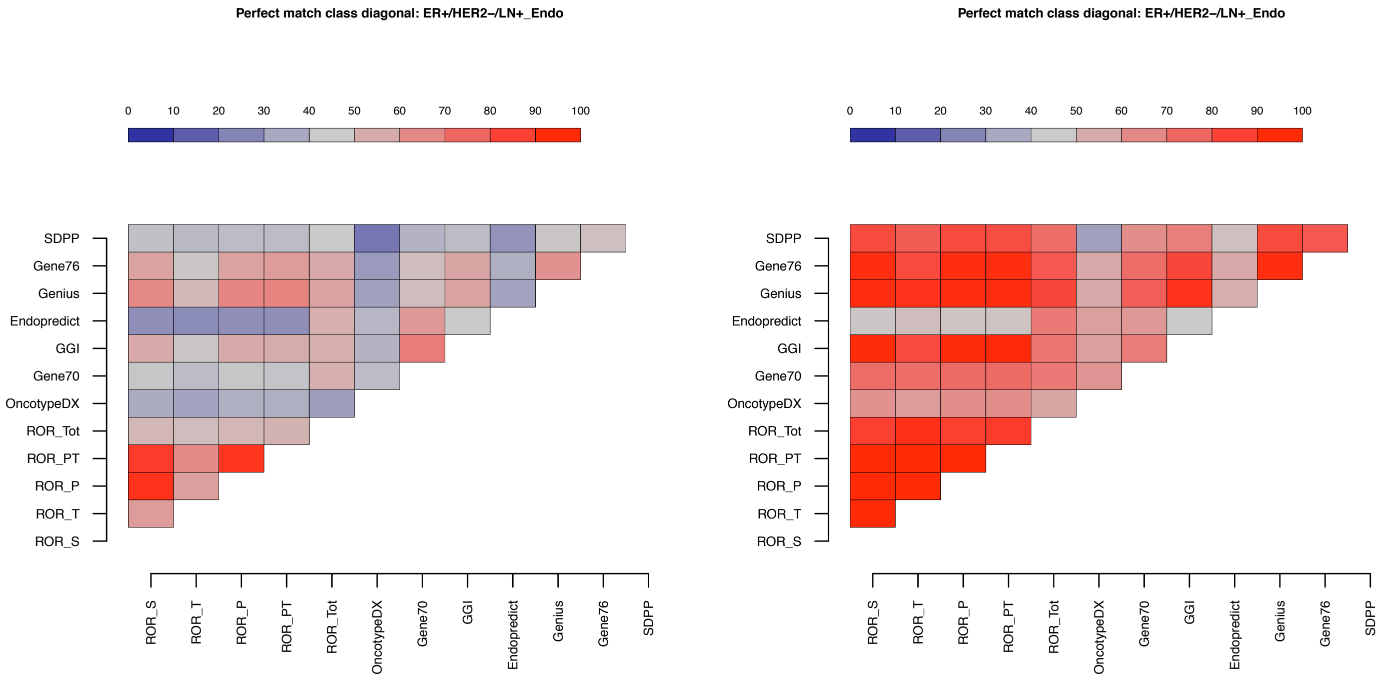

Perfect match class diagonal: ER+/HER2−/LN+_Endo
0 10 20 30 40 50 60 70 80 90 100
SDPP
Gene76
Genius
Endopredict
GGI
Gene70
OncotypeDX
ROR_Tot
ROR_PT
ROR_P
ROR_T
ROR_S
Perfect match class diagonal: ER+/HER2−/LN+_Endo

E)

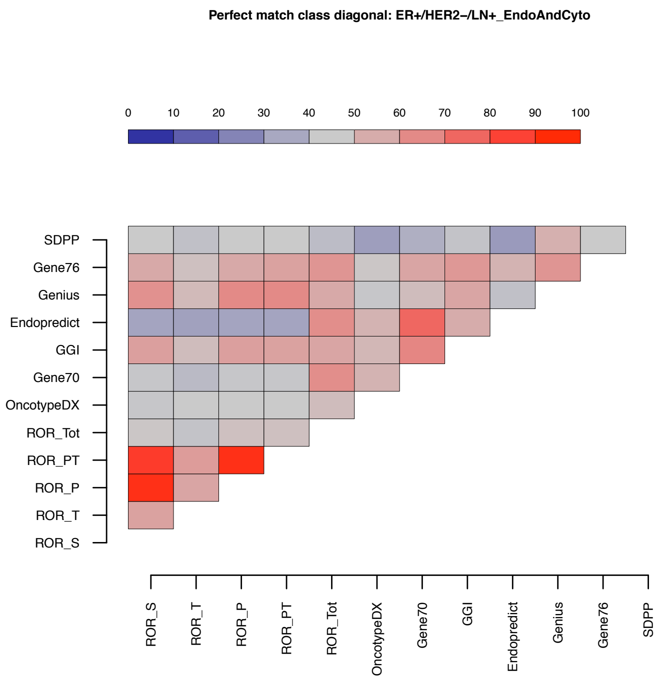
Perfect match class diagonal: ER+/HER2−/LN+_EndoAndCyto
0 10 20 30 40 50 60 70 80 90 100
SDPP
Gene76
Genius
Endopredict
GGI
Gene70
OncotypeDX
ROR_Tot
ROR_PT
ROR_P
ROR_T
ROR_S
ROR_S ROR_T ROR_P ROR_PT ROR_Tot OncotypeDX Gene70 GGI Endopredict Genius Gene76 SDPP

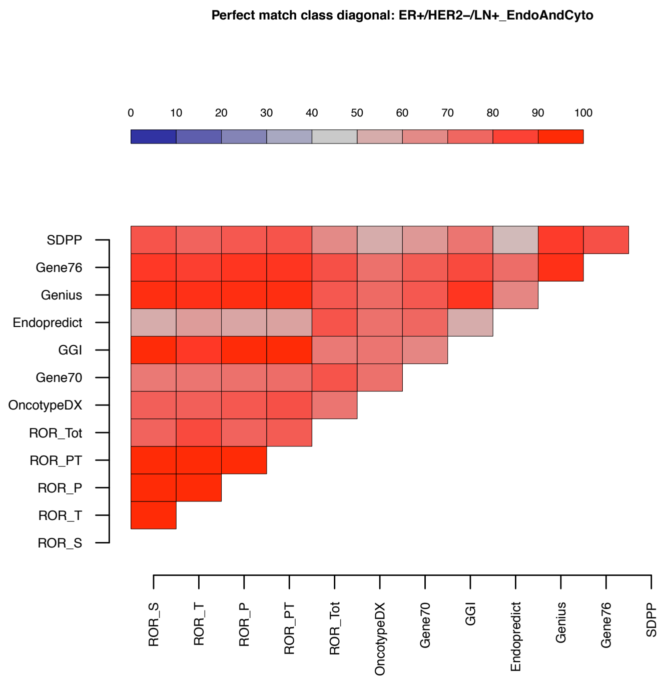
Perfect match class diagonal: ER+/HER2−/LN+_EndoAndCyto
0 10 20 30 40 50 60 70 80 90 100
SDPP
Gene76
Genius
Endopredict
GGI
Gene70
OncotypeDX
ROR_Tot
ROR_PT
ROR_P
ROR_T
ROR_S
ROR_S ROR_T ROR_P ROR_PT ROR_Tot OncotypeDX Gene70 GGI Endopredict Genius Gene76 SDPP

F)

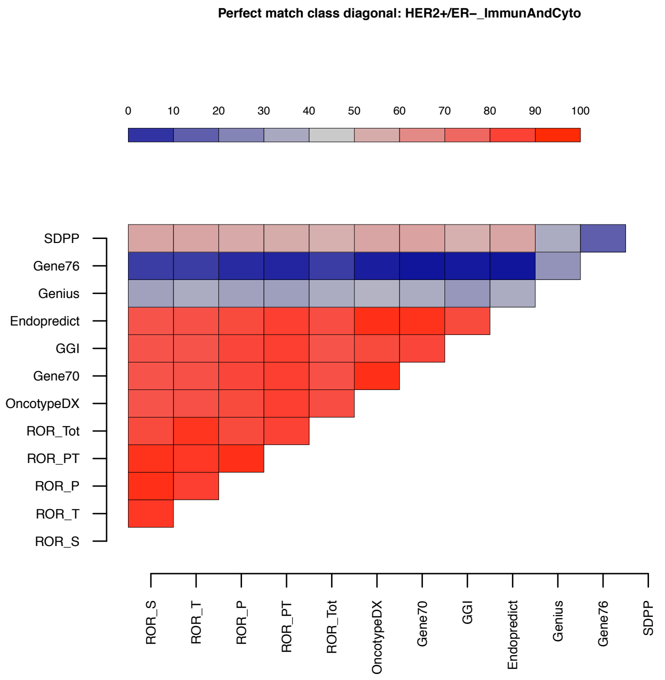
Perfect match class diagonal: HER2+/ER−_ImmunAndCyto
0 10 20 30 40 50 60 70 80 90 100
SDPP
Gene76
Genius
Endopredict
GGI
Gene70
OncotypeDX
ROR_Tot
ROR_PT
ROR_P
ROR_T
ROR_S
ROR_S ROR_T ROR_P ROR_PT ROR_Tot OncotypeDX Gene70 GGI Endopredict Genius Gene76 SDPP

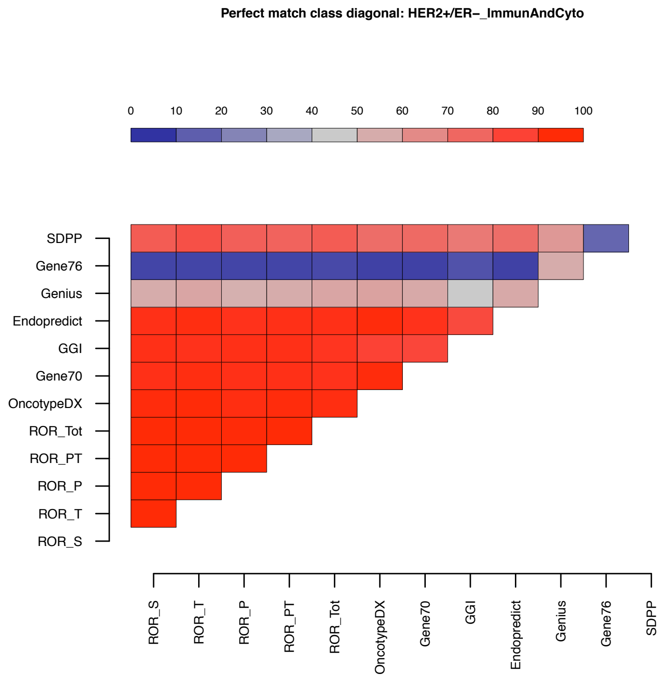
Perfect match class diagonal: HER2+/ER−_ImmunAndCyto
0 10 20 30 40 50 60 70 80 90 100
SDPP
Gene76
Genius
Endopredict
GGI
Gene70
OncotypeDX
ROR_Tot
ROR_PT
ROR_P
ROR_T
ROR_S
ROR_S ROR_T ROR_P ROR_PT ROR_Tot OncotypeDX Gene70 GGI Endopredict Genius Gene76 SDPP

G)

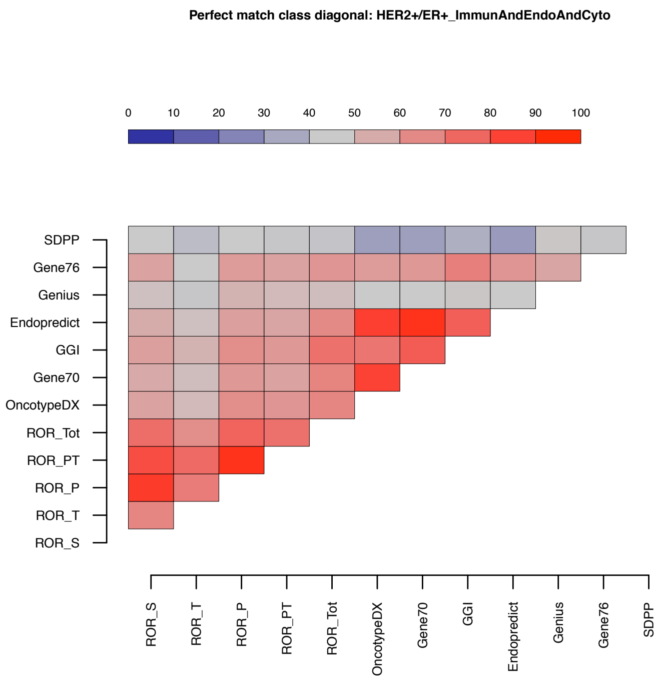
Perfect match class diagonal: HER2+/ER+_ImmunAndEndoAndCyto
0 10 20 30 40 50 60 70 80 90 100
SDPP
Gene76
Genius
Endopredict
GGI
Gene70
OncotypeDX
ROR_Tot
ROR_PT
ROR_P
ROR_T
ROR_S
ROR_S ROR_T ROR_P ROR_PT ROR_Tot OncotypeDX Gene70 GGI Endopredict Genius Gene76 SDPP

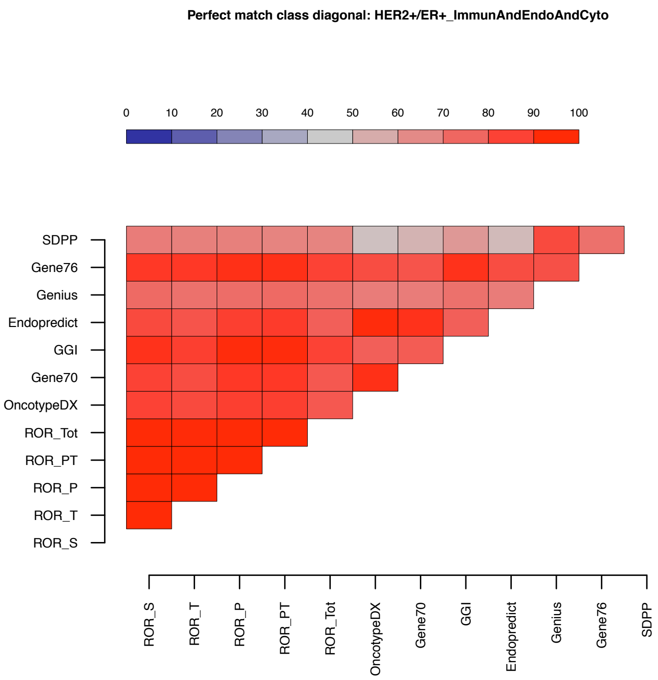
Perfect match class diagonal: HER2+/ER+_ImmunAndEndoAndCyto
0 10 20 30 40 50 60 70 80 90 100
SDPP
Gene76
Genius
Endopredict
GGI
Gene70
OncotypeDX
ROR_Tot
ROR_PT
ROR_P
ROR_T
ROR_S
ROR_S ROR_T ROR_P ROR_PT ROR_Tot OncotypeDX Gene70 GGI Endopredict Genius Gene76 SDPP

H)

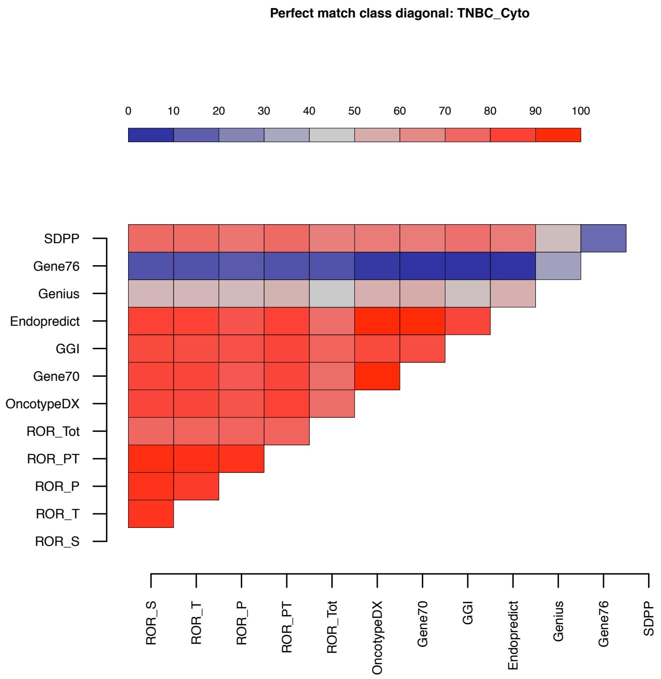
Perfect match class diagonal: TNBC_Cyto
0 10 20 30 40 50 60 70 80 90 100
SDPP
Gene76
Genius
Endopredict
GGI
Gene70
OncotypeDX
ROR_Tot
ROR_PT
ROR_P
ROR_T
ROR_S
ROR_S ROR_T ROR_P ROR_PT ROR_Tot OncotypeDX Gene70 GGI Endopredict Genius Gene76 SDPP

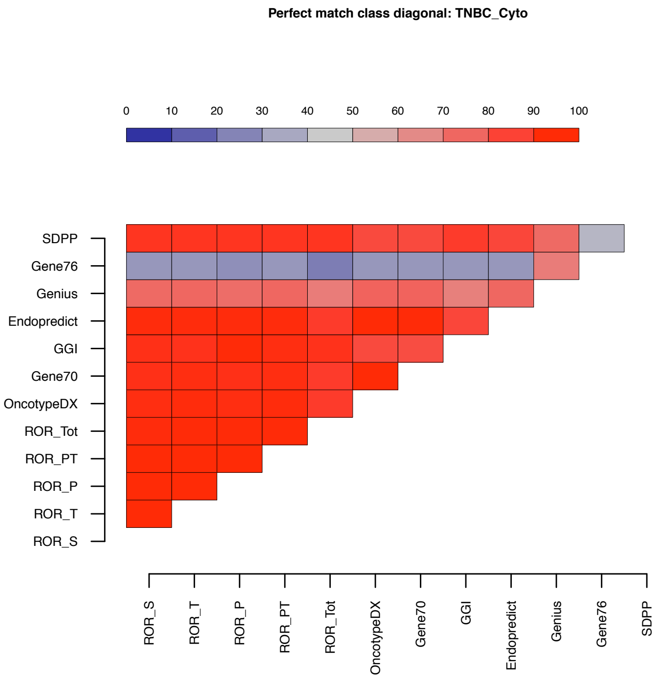
Perfect match class diagonal: TNBC_Cyto
0 10 20 30 40 50 60 70 80 90 100
SDPP
Gene76
Genius
Endopredict
GGI
Gene70
OncotypeDX
ROR_Tot
ROR_PT
ROR_P
ROR_T
ROR_S
ROR_S ROR_T ROR_P ROR_PT ROR_Tot OncotypeDX Gene70 GGI Endopredict Genius Gene76 SDPP

I)

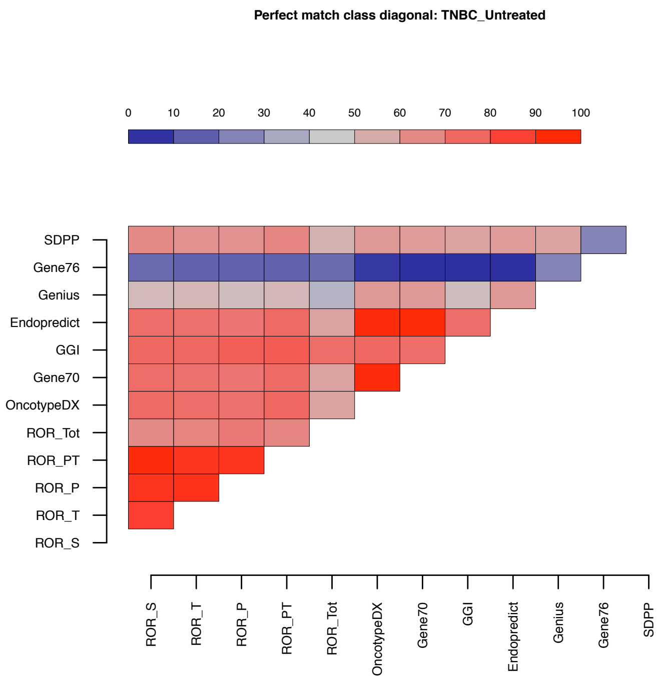
Perfect match class diagonal: TNBC_Untreated
0
10
20
30
40
50
60
70
80
90
100
SDPP
Gene76
Genius
Endopredict
GGI
Gene70
OncotypeDX
ROR_Tot
ROR_PT
ROR_P
ROR_T
ROR_S

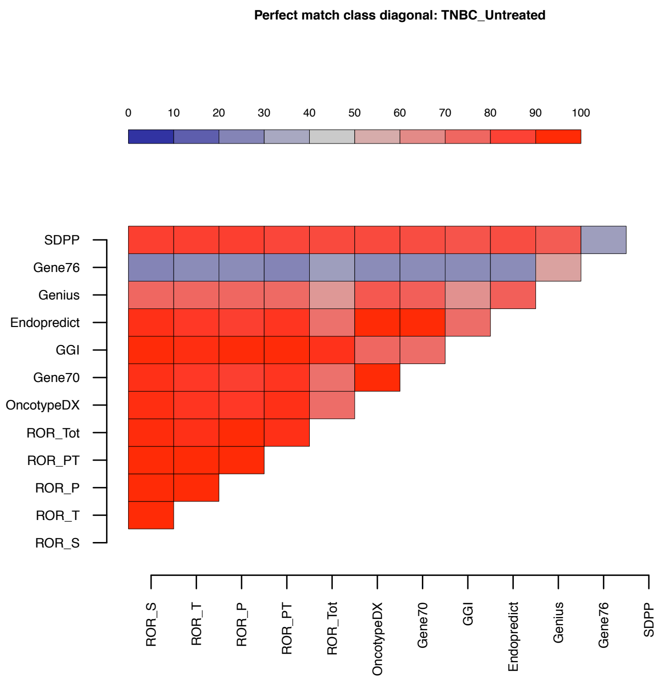
Perfect match class diagonal: TNBC_Untreated
0
10
20
30
40
50
60
70
80
90
100
SDPP
Gene76
Genius
Endopredict
GGI
Gene70
OncotypeDX
ROR_Tot
ROR_PT
ROR_P
ROR_T
ROR_S

Supplementary Figure S4. AIPS heatmaps of clinical assessment groups.

AIPS terms (heatmap, ternary data, high, intermediate, low) related to gene ontology terms were clustered for each subgroup using manhattan distance and complete linkage. (**A**) ER+/HER2-/LN- tumors including the three clinical assessment groups: i) untreated, ii) endocrine treatment only, and iii) ACT & endocrine. (**B**) ER+/HER2-/LN+ tumors including the two clinical assessment groups: i) endocrine treatment only, and ii) ACT & endocrine. (**C**) TNBC tumors including the two clinical assessment groups: i) ACT, and ii) untreated. (**D**) HER2+/ER- tumors with anti-HER2 & ACT treatment. (**E**) HER2+/ER+ tumors with anti-HER2 & ACT & endocrine therapy.

ACT: adjuvant chemotherapy.
OS: overall survival.

Legend for sub-panels concerning signature classes

Clinical Group
- ER+ / HER2- / LN-neg
- ER+ / HER2- / LN-pos
- HER2+ / ER-
- HER2+ / ER+
- TNBC
- NA

Treatment group
- Chemotherapy
- Endocrine
- Endocrine & Chemotherapy
- Anti-HER2 & Chemotherapy
- Anti-HER2 & Endocrine & Chemotherapy
- No adjuvant treatment
- NA

OS event
- 1
- 0

AIMS (PAM50)
- Basal
- LumA
- LumB
- Her2
- Normal
- unclassified

PAM50
- Basal
- LumA
- LumB
- Her2
- Normal
- unclassified

IC10
- 1
- 2
- 3
- 4
- 5
- 6
- 7
- 8
- 9
- 10

CIT
- Basal
- LumA
- LumB
- LumC
- mApo
- Normal

SCMOD2
- ER– / HER2–
- ER+ / HER2– High Proliferation
- ER+ / HER2– Low Proliferation
- HER2+

HDPP
- Cluster2
- Cluster3
- Cluster1

TNBCtype
- BL1
- BL2
- IM
- LAR
- M
- MSL
- UNS

ROR-S
- High-risk
- Intermediate-risk
- Low-risk

ROR-T
- High-risk
- Intermediate-risk
- Low-risk

ROR-P
- High-risk
- Intermediate-risk
- Low-risk

ROR-PT
- High-risk
- Intermediate-risk
- Low-risk

ROR-Tot
- High-risk
- Intermediate-risk
- Low-risk

Oncotype DX
- High-risk
- Intermediate-risk
- Low-risk

Gene70
- High-risk
- Low-risk

GGI
- High-risk
- Low-risk

Endopredict
- High-risk
- Low-risk

Genius
- High-risk
- Intermediate-risk
- Low-risk

Gene76
- High-risk
- Intermediate-risk
- Low-risk

SDPP
- High-risk
- Intermediate-risk
- Low-risk

AIPS expression
- High
- Intermediate
- Low

A) ER+/HER2-/LN- : n=1563

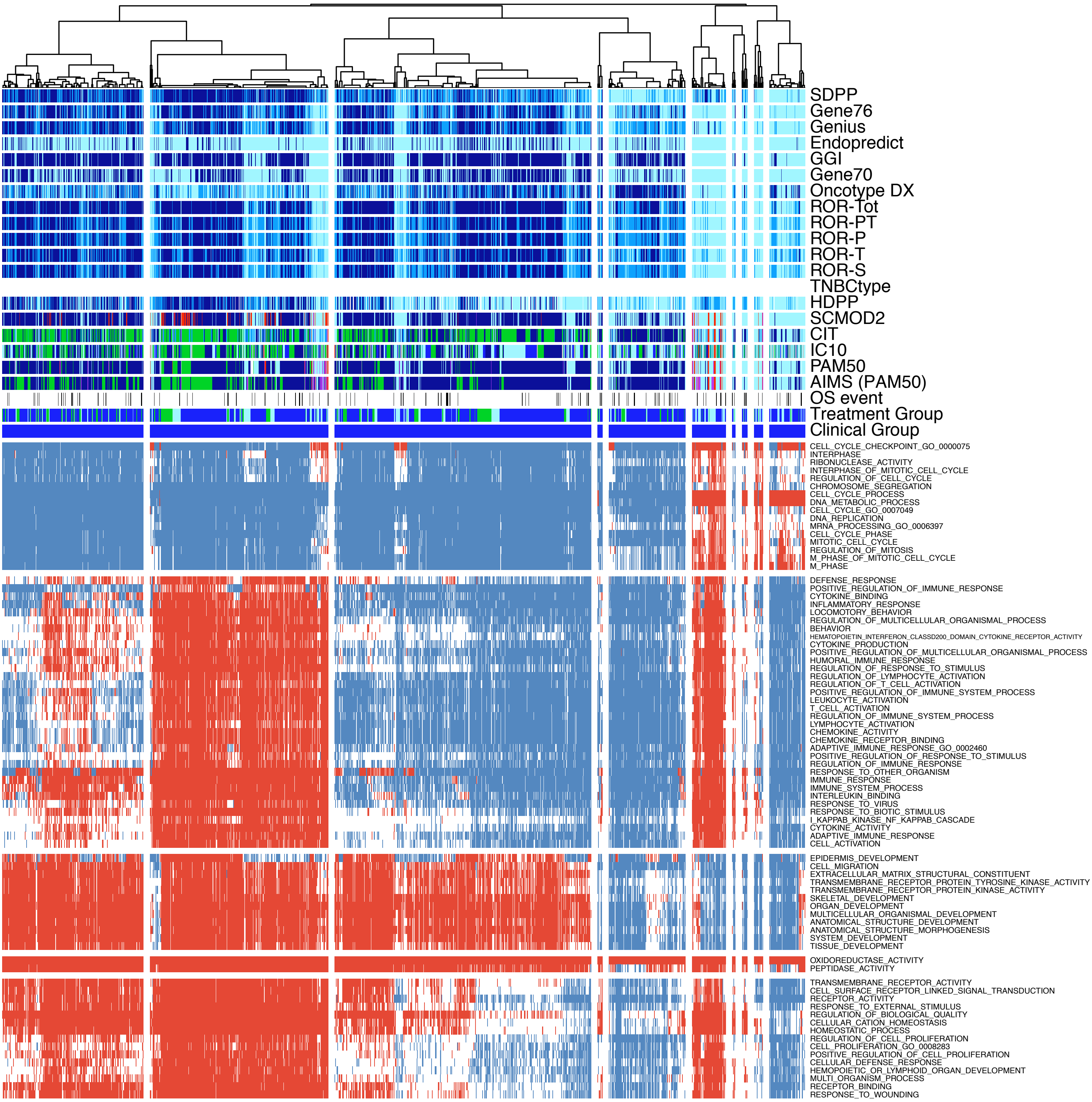

B) ER+/HER2-/LN+ : n=862

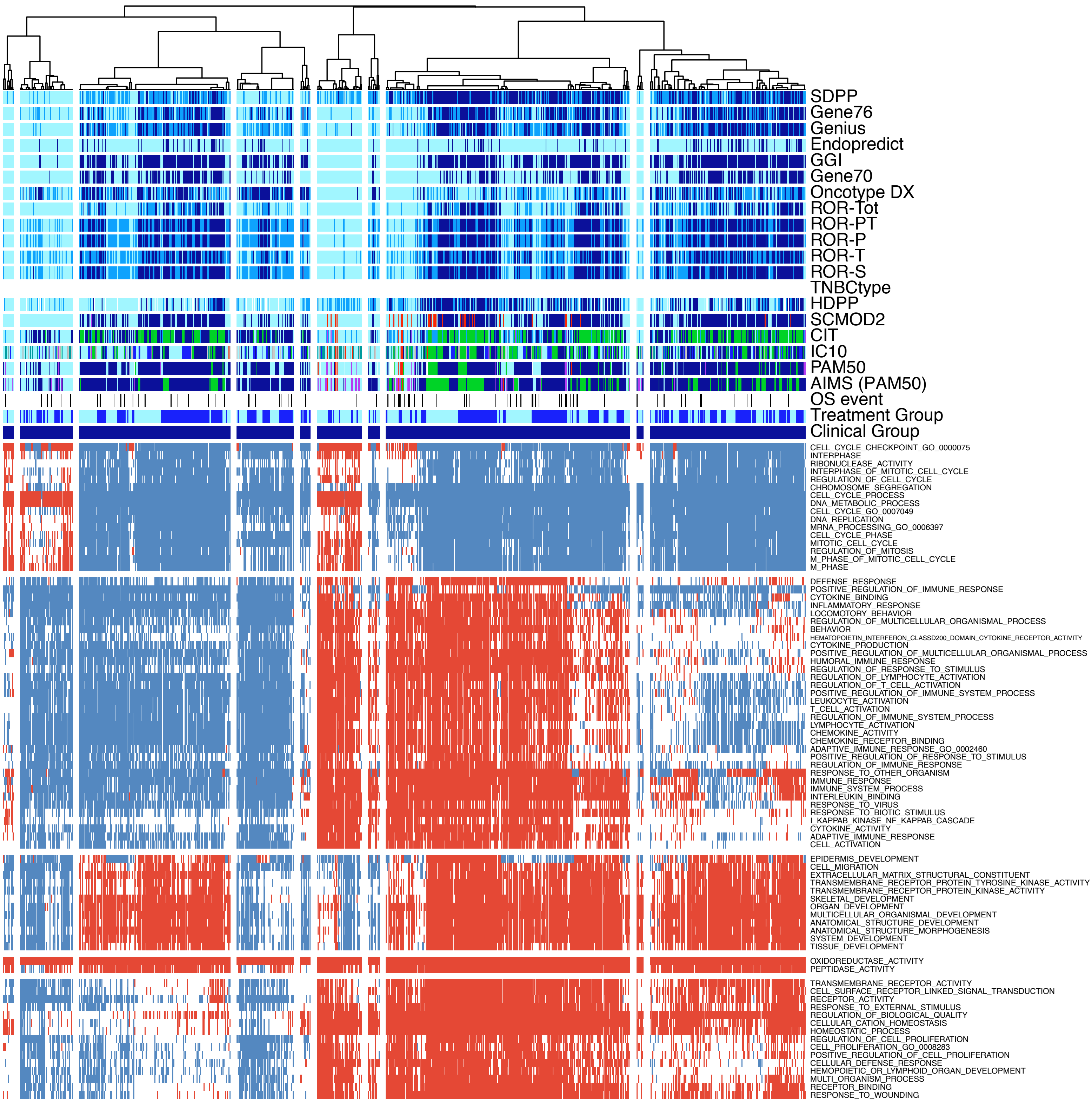

C) TNBC: n=321

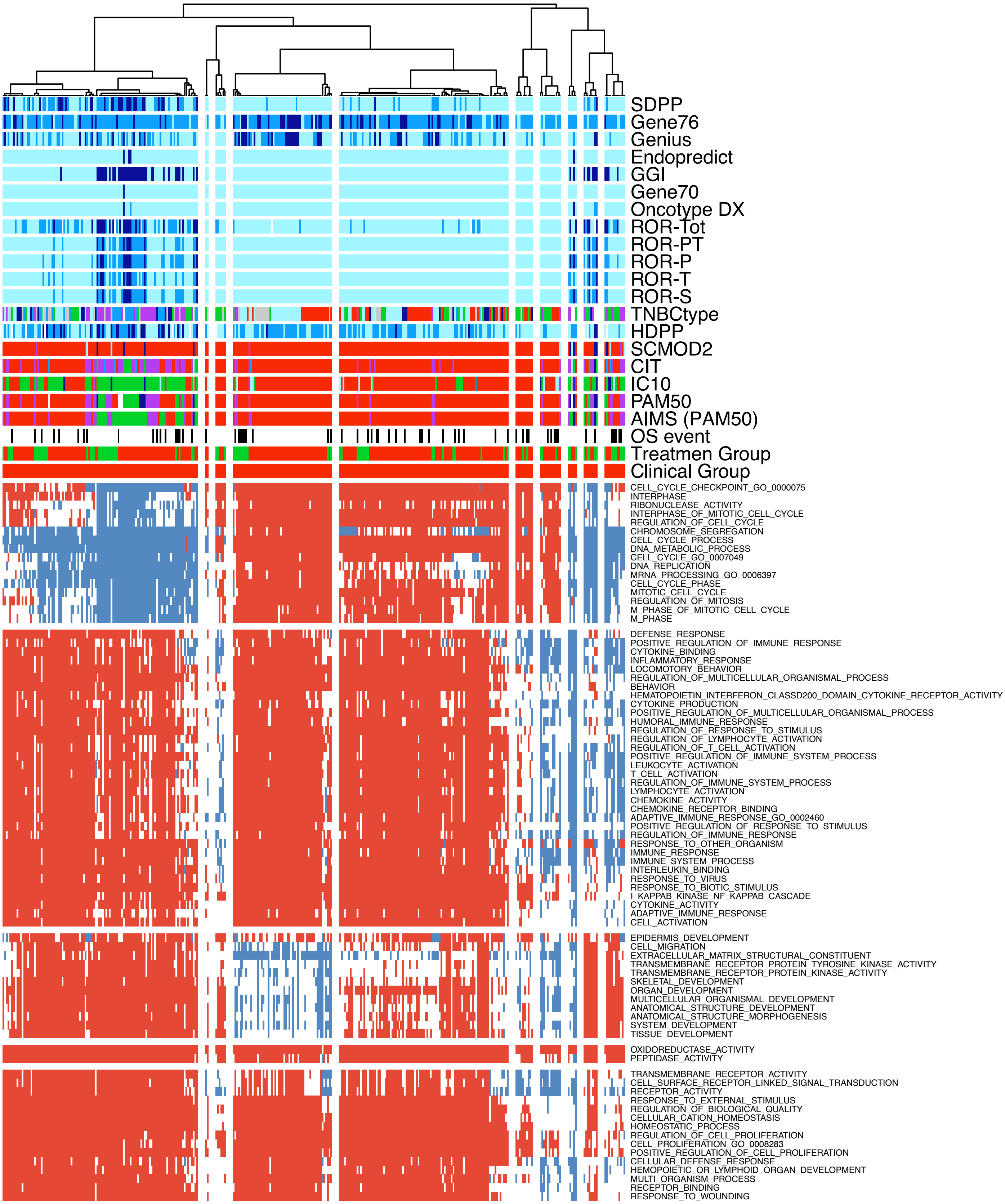
SDPP
Gene76
Genius
Endopredict
GGI
Gene70
Oncotype DX
ROR-Tot
ROR-PT
ROR-P
ROR-T
ROR-S
TNBCtype
HDPP
SCMOD2
CIT
IC10
PAM50
AIMS (PAM50)
OS event
Treatmen Group
Clinical Group
CELL_CYCLE_CHECKPOINT_GO_0000075
INTERPHASE
RIBONUCLEASE_ACTIVITY
INTERPHASE_OF_MITOTIC_CELL_CYCLE
REGULATION_OF_CELL_CYCLE
CHROMOSOME_SEGREGATION
CELL_CYCLE_PROCESS
DNA_METABOLIC_PROCESS
CELL_CYCLE_GO_0007049
DNA_REPLICATION
MRNA_PROCESSING_GO_0006397
CELL_CYCLE_PHASE
MITOTIC_CELL_CYCLE
REGULATION_OF_MITOSIS
M_PHASE_OF_MITOTIC_CELL_CYCLE
M_PHASE
DEFENSE_RESPONSE
POSITIVE_REGULATION_OF_IMMUNE_RESPONSE
CYTOKINE_BINDING
INFLAMMATORY_RESPONSE
LOCOMOTORY_BEHAVIOR
REGULATION_OF_MULTICELLULAR_ORGANISMAL_PROCESS
BEHAVIOR
HEMATOPOIETIN_INTERFERON_CLASSD200_DOMAIN_CYTOKINE_RECEPTOR_ACTIVITY
CYTOKINE_PRODUCTION
POSITIVE_REGULATION_OF_MULTICELLULAR_ORGANISMAL_PROCESS
HUMORAL_IMMUNE_RESPONSE
REGULATION_OF_RESPONSE_TO_STIMULUS
REGULATION_OF_LYMPHOCYTE_ACTIVATION
REGULATION_OF_T_CELL_ACTIVATION
POSITIVE_REGULATION_OF_IMMUNE_SYSTEM_PROCESS
LEUKOCYTE_ACTIVATION
T_CELL_ACTIVATION
REGULATION_OF_IMMUNE_SYSTEM_PROCESS
LYMPHOCYTE_ACTIVATION
CHEMOKINE_ACTIVITY
CHEMOKINE_RECEPTOR_BINDING
ADAPTIVE_IMMUNE_RESPONSE_GO_0002460
POSITIVE_REGULATION_OF_RESPONSE_TO_STIMULUS
REGULATION_OF_IMMUNE_RESPONSE
RESPONSE_TO_OTHER_ORGANISM
IMMUNE_RESPONSE
IMMUNE_SYSTEM_PROCESS
INTERLEUKIN_BINDING
RESPONSE_TO_VIRUS
RESPONSE_TO_BIOTIC_STIMULUS
I_KAPPAB_KINASE_NF_KAPPAB_CASCADE
CYTOKINE_ACTIVITY
ADAPTIVE_IMMUNE_RESPONSE
CELL_ACTIVATION
EPIDERMIS_DEVELOPMENT
CELL_MIGRATION
EXTRACELLULAR_MATRIX_STRUCTURAL_CONSTITUENT
TRANSMEMBRANE_RECEPTOR_PROTEIN_TYROSINE_KINASE_ACTIVITY
TRANSMEMBRANE_RECEPTOR_PROTEIN_KINASE_ACTIVITY
SKELETAL_DEVELOPMENT
ORGAN_DEVELOPMENT
MULTICELLULAR_ORGANISMAL_DEVELOPMENT
ANATOMICAL_STRUCTURE_DEVELOPMENT
ANATOMICAL_STRUCTURE_MORPHOGENESIS
SYSTEM_DEVELOPMENT
TISSUE_DEVELOPMENT
OXIDOREDUCTASE_ACTIVITY
PEPTIDASE_ACTIVITY
TRANSMEMBRANE_RECEPTOR_ACTIVITY
CELL_SURFACE_RECEPTOR_LINKED_SIGNAL_TRANSDUCTION
RECEPTOR_ACTIVITY
RESPONSE_TO_EXTERNAL_STIMULUS
REGULATION_OF_BIOLOGICAL_QUALITY
CELLULAR_CATION_HOMEOSTASIS
HOMEOSTATIC_PROCESS
REGULATION_OF_CELL_PROLIFERATION
CELL_PROLIFERATION_GO_0008283
POSITIVE_REGULATION_OF_CELL_PROLIFERATION
CELLULAR_DEFENSE_RESPONSE
HEMOPOIETIC_OR_LYMPHOID_ORGAN_DEVELOPMENT
MULTI_ORGANISM_PROCESS
RECEPTOR_BINDING
RESPONSE_TO_WOUNDING

D) HER2+/ER- anti-HER2 & ACT: n=111

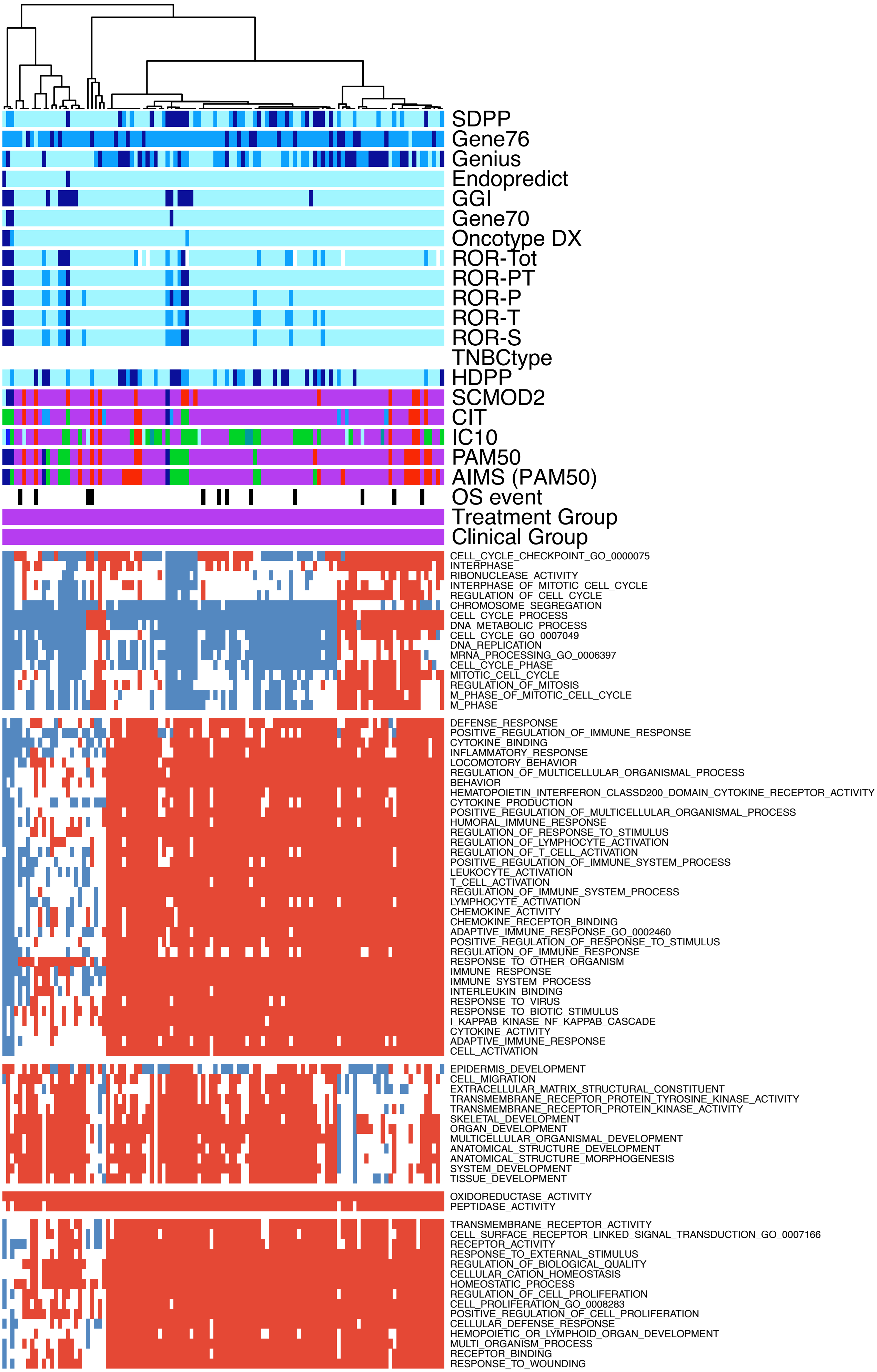

E) HER2+/ER+ anti-HER2 & ACT & endocrine: n=239

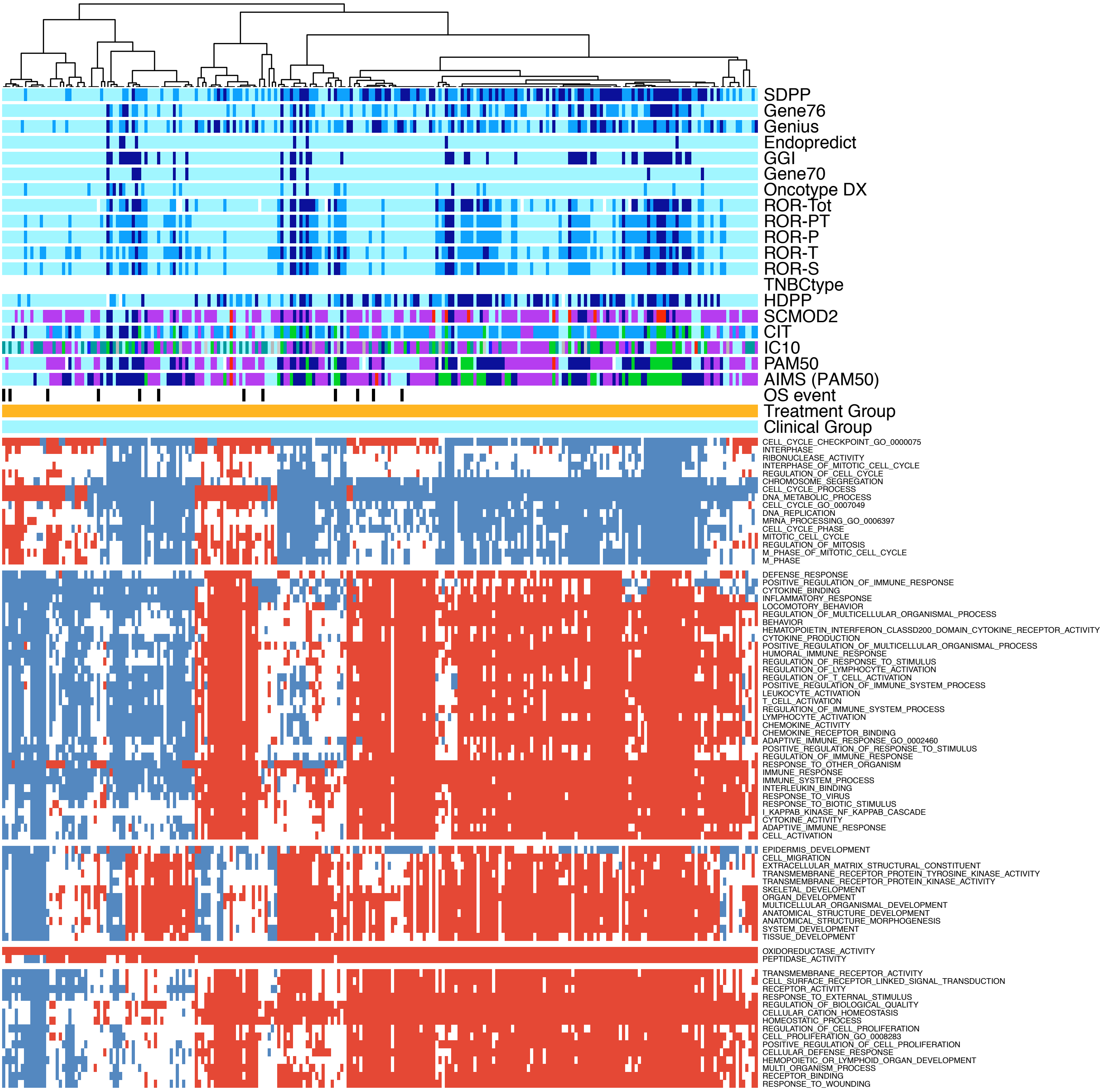

Supplementary Figure S5. Risk prediction signature consensus voting in luminal clinical assessment groups

For each clinical assessment group and sample the number of low-risk predictions by eight risk prediction signatures (ROR-S, Oncotype DX, Gene70, Gene76, SDPP, GGI, Endopredict, Genius) was summarized. The extreme groups (0-1 low-risk predictions versus 7-8 low-risk predictions) were contrasted against each other in a Kaplan-Meier analysis for clinical assessment groups. For each subgroup the number of cases and the proportion of the total respective assessment group is shown. (**A**) ER+/HER2-/LN+ with endocrine treatment. (**B**) ER+/HER2-/LN+ with ACT & endocrine treatment. (**C**) ER+/HER2-/LN- with endocrine treatment. (**D**) ER+/HER2-/LN- untreated.
Panel legends: Endo: endocrine therapy. ACT: adjuvant chemotherapy.

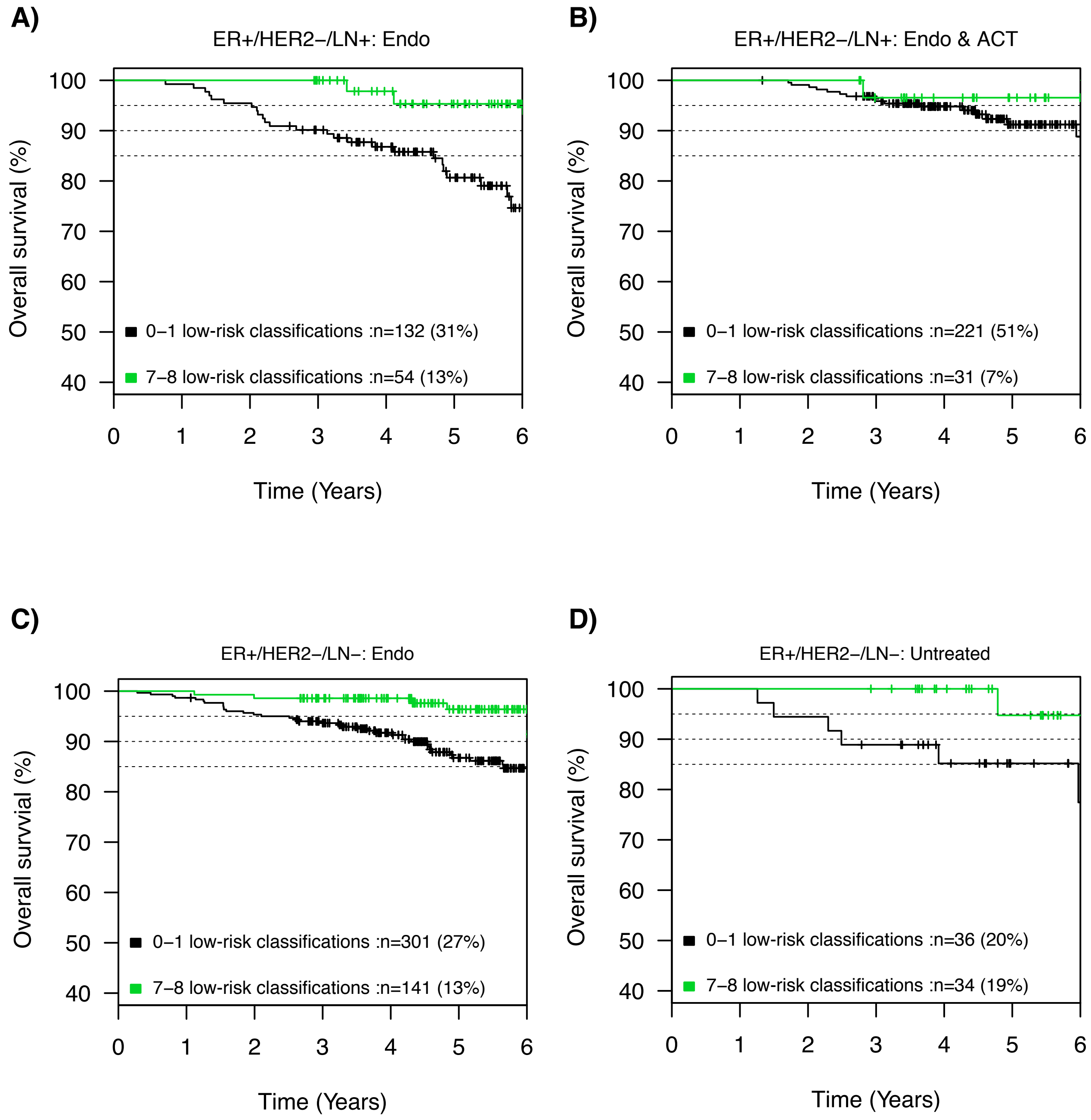

Supplement: Supplementary file 1 — Supplementary Information [file 41598_2019_48570_MOESM1_ESM.pdf]
